# Translational Modeling of Chloroquine and Hydroxychloroquine Dosimetry in Human Airways for Treating Viral Respiratory Infections

**DOI:** 10.1007/s11095-021-03152-3

**Published:** 2022-01-09

**Authors:** Aditya R. Kolli, Florian Calvino-Martin, Julia Hoeng

**Affiliations:** PMI R&D, Philip Morris Products S.A., Quai Jeanrenaud 5, CH-2000 Neuchâtel, Switzerland

**Keywords:** antiviral, pulmonary, pharmacokinetics, influenza, coronavirus

## Abstract

**Purpose:**

Chloroquine and hydroxychloroquine are effective against respiratory viruses *in vitro*. However, they lack antiviral efficacy upon oral administration. Translation of *in vitro* to *in vivo* exposure is necessary for understanding the disconnect between the two to develop effective therapeutic strategies.

**Methods:**

We employed an *in vitro* ion-trapping kinetic model to predict the changes in the cytosolic and lysosomal concentrations of chloroquine and hydroxychloroquine in cell lines and primary human airway cultures. A physiologically based pharmacokinetic model with detailed respiratory physiology was used to predict regional airway exposure and optimize dosing regimens.

**Results:**

At their reported *in vitro* effective concentrations in cell lines, chloroquine and hydroxychloroquine cause a significant increase in their cytosolic and lysosomal concentrations by altering the lysosomal pH. Higher concentrations of the compounds are required to achieve similar levels of cytosolic and lysosomal changes in primary human airway cells *in vitro*. The predicted cellular and lysosomal concentrations in the respiratory tract for *in vivo* oral doses are lower than the *in vitro* effective levels. Pulmonary administration of aerosolized chloroquine or hydroxychloroquine is predicted to achieve high bound *in vitro*-effective concentrations in the respiratory tract, with low systemic exposure. Achieving effective cytosolic concentrations for activating immunomodulatory effects and adequate lysosomal levels for inhibiting viral replication could be key drivers for treating viral respiratory infections.

**Conclusion:**

Our analysis provides a framework for extrapolating *in vitro* effective concentrations of chloroquine and hydroxychloroquine to *in vivo* dosing regimens for treating viral respiratory infections.

**Graphical abstract:**

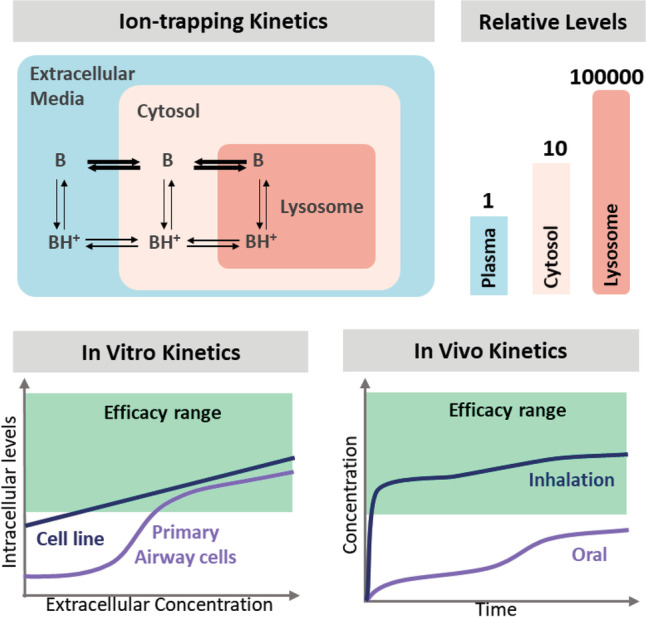

**Supplementary Information:**

The online version contains supplementary material available at 10.1007/s11095-021-03152-3.

## Introduction

Respiratory viruses are transmitted from person to person and cause diseases in humans, some of which have high morbidity and mortality. Common respiratory viruses among humans include adenoviruses, bocaviruses, coronaviruses, metapneumovirus, influenza viruses, parainfluenza viruses, respiratory syncytial virus, and rhinoviruses [1, 2]. The primary and most susceptible sites for viral infection are the epithelial cells lining the nasopharynx to bronchial airways [3], and cause common cold or exacerbation of other respiratory diseases [2, 4].

Successful entry and release of a virus into epithelial cells is a tightly regulated process, requiring events such as virus binding to cell surface receptors and physiological environmental cues such as acidic pH [5]. For example, Rhinoviruses bind to ICAM-1 (intracellular adhesion molecule-I), LDLR (low-density lipoprotein receptor), or CDHR3 (cadherin related family member 3) for cellular entry [6], and coronaviruses bind to the surface angiotensin-converting enzyme 2 (ACE2) receptor [7]. The binding of viruses to cellular surface receptors and entry into epithelial cells is efficient under acidic environments [8]; the viruses may undergo endocytosis or non-endocytic fusion to enter cells. During endocytosis, the endolysosomal pH gradually drops from 6.8 to 6.1 in early endosomes, from 6.0 to 4.8 in late endosomes, and from 5.0 to 4.5 in lysosomes [9], creating a favorable environment for the virus to undergo post-translational modifications and enter the host cell. Earlier studies have shown that adenoviruses [10], coronaviruses [11], and influenza viruses [12] require acidified endolysosomes for viral infection. Compounds such as bafilomycin A1, ammonium chloride, chloroquine (CQ), and hydroxychloroquine (HCQ) have been shown to lower endolysosomal acidification and inhibit viral replication *in vitro* [13–16].

In viral respiratory infections (VRI), clinical symptoms result from an elaborate activation of pro-inflammatory mediators by the epithelial cells lining the respiratory tract [17]. The severity of respiratory symptoms can be correlated to the elevated levels of cytokines in plasma and nasal secretions [18–20]. Respiratory viruses such as rhinoviruses also activate the production of potent pro-inflammatory mediators (chemokines) such as interferon gamma-induced protein 10 (IP-10) and RANTES [21]. CQ and HCQ, at an *in vitro* concentration of 10 μM, inhibit endosomal TLR- (toll-like receptor) and cGAS- (cytoplasmic cyclic guanosine monophosphate–adenosine monophosphate synthase) mediated activation of pro-inflammatory cytokines such as TNF-α, IL-1β, IL-6, and INF-γ [22].

**Fig. 1:**
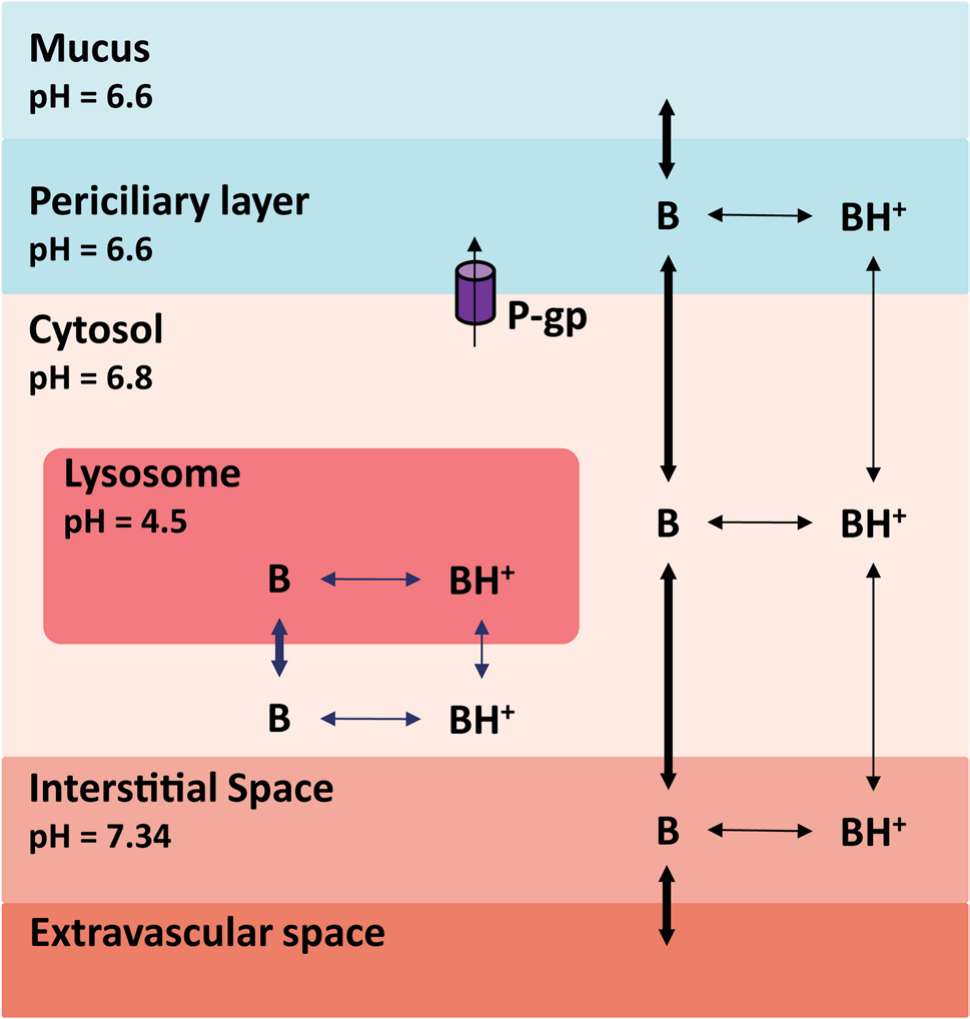
Schematic of compound kinetics and ion-trapping of chloroquine and hydroxychloroquine across the airways. The unprotonated base (B) moves more rapidly than the protonated form (BH^+^). P-gp, P-glycoprotein transporter.

CQ and HCQ are well-known anti-malarial drugs which were studied as antiviral agents for treating coronavirus, ebola, human immunodeficiency virus, and influenza virus infections [23, 24]. Both compounds are diprotic bases with lysosomotropic properties [23], and Fig. 1 shows a schematic of their cellular distribution. Their unionized forms (B) can diffuse rapidly across cell membranes and organelles, whereas their protonated forms (BH+) move slowly. In acidic environments, the unionized forms of the bases become protonated and trapped. The magnitude of accumulation in organelles depends on the physiochemical properties and pH of the environment. CQ and HCQ can assume monoprotonated and biprotonated forms, magnifying the lysosomal ion trapping by more than 60,000-fold beyond that in the extracellular environment [25, 26].

Several *in vivo* studies have evaluated the efficacy of orally administered CQ and HCQ against respiratory viruses [27, 28]. Despite its promising *in vitro* results against the influenza virus, 12.5 mg/kg oral CQ did not prevent infection in mouse and ferret animal models in a previous study [27]. A randomized clinical study by Paton *et al*. also found that 500 mg/day oral dose of chloroquine phosphate (310 mg/day CQ base) does not prevent influenza [29]. However, CQ was found to be effective against coronavirus strain OC43 in mice at a high oral dose of 15 mg/kg [30]. Emergency-use authorization and large-scale clinical trials for oral dosing of CQ and HCQ have been implemented in various countries for treating severe acute respiratory syndrome coronavirus 2 (SARS-CoV-2) infection [31–33]. Initial studies have shown no improvement in morbidity, as oral administration of these drugs does not lower the viral load [34–36].

The clinical efficacy of CQ and HCQ in treating VRIs remains unclear and could be linked to *in vivo* regional concentrations in the respiratory tract [37]. Hence, there is a need to translate *in vitro* to *in vivo* kinetics in order to obtain an understanding of the regional drug distribution in the respiratory tract. In this study, we applied an ion-trapping model to predict the cytosolic and endolysosomal concentrations of CQ and HCQ *in vitro*. A physiologically based pharmacokinetic (PBPK) model for each of these compounds was applied to predict the cytosolic and lysosomal concentrations along the respiratory tract for different routes of administration [38].

## Methods

The *in vitro* model for cellular uptake and lysosomal ion trapping was implemented as described by Trapp *et al*. [25]. Briefly, the total net diffusive flux (*J*_*net*_) between the compartments is the sum of the diffusive flux of neutral species and ionic species (Eq. 1).1where *P* is the permeability; *a* is the activity of the compound; *N* = *ɀEF*/(*RT*); *ɀ* is the electric charge (0 for neutral and + 1 and + 2 for ionic species); *F* is the Faraday constant; *E* is the membrane potential (−70 mV for the cell and 100 mV for a lysosome); *R* is the real gas constant; and *T* is the temperature. The subscripts indicate the fractions of neutral (*neu*) and ionic (*ion*) species present inside (*in*) and outside (*out*) the compartments.

Permeability (*P*) of the compound is calculated based on the diffusion coefficient (*DS*), partition coefficient (*K*) and membrane thickness (*Δx*) as shown in Eq. 2 [39]. However, Eq.2 can be written in a log-linear relationship (Eq.3) by approximating *K* to compound specific lipophilicity (*Kow*), and *Δs* for capturing the organic compound-specific diffusion across a 50 nm membrane [39] to determine the permeability of neutral species (*P*_*neu*_). Considering that ionized species travel slowly across membranes, the permeability for ionic species (*P*_*ion*_) was set to be lower than neutral species [39] (Eq. 4).

2 $$P=\frac{DS\ast K}{\varDelta x}$$

3 $${P}_{neu}={10}^{\log Kow-\varDelta s}$$

4

The neutral (*f*_*neu*_) and ionic () fractions of the drug were determined on the basis of a water fraction (*W*) of 0.95 g/g, lipid binding (*L*) of 0.05 g/g, sorption coefficients (*K*), and ionic activity coefficients (*γ*) [25], as shown in Eq. 5 and 6.56where the ionic activity coefficient for a species with charge 0 is 1.23, +1 is 0.74, and + 2 is 0.3 [25]. The ratio of ionic fractions (${D}_{io{n}_1}$ and ${D}_{io{n}_2}$) between the species in the compartment were calculated by using eqs. 7 and 8.

7 $${D}_{io{n}_1}=\frac{10^{\left(p{K}_{a1}- pH\right)}}{1+{10}^{\left(p{K}_{a1}- pH\right)}+{10}^{\left(p{K}_{a1}- pH\right)}+{10}^{\left(p{K}_{a2}- pH\right)}}$$

8 $${D}_{io{n}_2}=\frac{10^{\left(p{K}_{a1}- pH\right)}+{10}^{\left(p{K}_{a2}- pH\right)}}{1+{10}^{\left(p{K}_{a1}- pH\right)}+{10}^{\left(p{K}_{a1}- pH\right)}+{10}^{\left(p{K}_{a2}- pH\right)}}$$

The sorption coefficients () were determined using lipophilicity and lipid binding as described in Eqs. 9 and 10. The lysosomal pH changes were modeled using Eq. 11.910

11 $$p{H}_{lys}=p{H}_{lys,t=0}-\frac{C_{lys}}{\beta }$$

where *pH*_*lys*, *t* = 0_ is the initial pH of the lysosome; *C*_*lys*_ is the concentration of drug in the lysosome, and *β* is the lysosomal buffering capacity [40, 41]. The differential equations describing the changes in concentrations (*C*) in the extracellular (*out*), cytosol (*cyt*), and lysosomal (*lys*) compartments are as follows.

12 $$\frac{\mathrm{d}}{\mathrm{d}\mathrm{t}}{\mathrm{C}}_{\mathrm{out}}=\frac{1}{{\mathrm{V}}_{\mathrm{out}}}\left(-{\mathrm{SA}}_{\mathrm{cyt}}\ \left({J}_{\mathrm{out}-\mathrm{cyt}}-{J}_{\mathrm{cyt}-\mathrm{out}}\right)\right)$$

13 $$\frac{\mathrm{d}}{\mathrm{d}\mathrm{t}}{\mathrm{C}}_{\mathrm{cyt}}=\frac{1}{{\mathrm{V}}_{\mathrm{cyt}}-{V}_{lys}}\left({\mathrm{SA}}_{\mathrm{cyt}}\ \left({J}_{\mathrm{out}-\mathrm{cyt}}-{J}_{\mathrm{cyt}-\mathrm{out}}\right)-{\mathrm{SA}}_{\mathrm{lys}}\ \left({J}_{\mathrm{cyt}-\mathrm{lys}}-{J}_{\mathrm{lys}-\mathrm{cyt}}\right)\right)$$

14 $$\frac{\mathrm{d}}{\mathrm{d}\mathrm{t}}{\mathrm{C}}_{\mathrm{lys}}=\frac{1}{{\mathrm{V}}_{\mathrm{lys}}}\left(\ {\mathrm{SA}}_{\mathrm{lys}}\ \left({J}_{\mathrm{cyt}-\mathrm{lys}}-{J}_{\mathrm{lys}-\mathrm{cyt}}\right)\ \right)$$

where *SA* is surface area and *V* is volume. The model parameters are listed in Table I, and the model code is provided in the Supplementary Online Resource.

**Table I:** List of the Generic and *In Vitro* Model Parameters [38]

Generic model parameters											
Drug	pKa1	pKa2	Octanal-water partition, *logKow*	Molecular weight		Blood-plasma ratio, R_bp_	Unbound protein fraction, f_u_		Diffusion coefficient, *DC* (cm^2^/s)	Diffusivity factor, Δs	
CQ	9.4	8.2	4.69	320		8.0	0.6		6.49E-11	7.4 ^a^	
HCQ	9.67	8.27	3.89	336		7.2	0.45		6.42E-11	6.8 ^a^	
In vitro model parameters											
		*Isolated rat hepatocytes* [42]			*Cell line*			*Primary human airway culture* [38]			
Compartment		Diameter, *d* (m)	Volume, *V*	pH	Diameter, *d* (m)	Volume, *V*	pH	Surface area, *SA* (m^2^)	Thickness, *T* (m)	Volume, *V*	pH
Cell		1.76E-5	2% ^b^	7.2 ^e^	1.7E-5 ^d^	1.93E-15 m^3^	7.2 ^e^	0.33E-4	4E-5		7.2
Lysosome		0.5E-6	1.5% ^c^	4.7	0.5E-6	1% ^c^	4.5 ^e^			8% ^c^	4.5
Mucus								0.33E-4	3E-6		7.2
Periciliary layer								0.33E-4	3E-6		7.2
Extracellular or basal			98%							0.75 mL	7.4

^a^estimated, ^b^ packed volume, ^c^ cellular volume, ^d^ ATCC® CRL-1586™, ^e^ fixed

We have recently developed a PBPK model for CQ and HCQ, which consists of 16 tissue compartments [38]. In this model, the lysosomal compartment is nested in each tissue compartment, and the kinetics of lysosomal trapping are implemented in a similar form as in the *in vitro* model [25, 41]. The non-lysosomal tissue (*C*_*tis*_) and lysosomal (${C}_{tis_{lys}}$) concentrations are described using a general mass balance equation (Eqs. 15 and 16).

15 $$\frac{d}{dt}{C}_{tis}=\frac{1}{V_{tis}-{V}_{tis_{lys}}}\left({Q}_{tis}\left({C}_{art}-\frac{C_{tis}\ast {R}_{bp}}{f_u\ast {K}_{ptu}}\right)\hbox{--} S{A}_{tis_{lys}}\left({J}_{tis- lys}\hbox{--} {J}_{lys- tis}\right)\right)$$

16 $$\frac{d}{dt}{C}_{tis_{lys}}=\frac{1}{V_{tis_{lys}}}\left(S{A}_{tis_{lys}}\left({J}_{tis- lys}\hbox{--} {J}_{lys- tis}\right)\right)$$

where *C*_*art*_ is the arterial blood concentration; *Q* is the blood flow rate; *V* is volume; $S{A}_{tis_{lys}}$ is the total surface area of the lysosomes; *K*_*ptu*_ is the tissue–plasma partition coefficient; *R*_*bp*_ is the blood-to-plasma ratio; and *f*_*u*_ is the unbound fraction in plasma. The model for the respiratory tract was adopted from Sarangapani *et al*. [43]. The four regions of respiratory tract were the upper airways (UA), conducting airways (CA), transitional airways (TA), and pulmonary alveolar region (PA) [43]. Each respiratory tract region was further divided to represent the mucus, periciliary layer, cytosol, and interstitial and vascular compartments. The lysosomal compartment was nested in the cytosol [38]. To mimic human physiological conditions, a pH of 6.6 for periciliary layer, 6.8 for cytosol and 4.5 for lysosomes was used [38]. The mucociliary clearance rates from Asgharian *et al*. [44] and active transport via the P-gp efflux transporter from Price *et al*. [45] were included in the model. The model was constructed in R Studio version 3.5.1 (RStudio, Boston, MA, USA) by using the *mrgsolve* package version 0.8.12 [46]. The model parameters are listed in Table II.

**Table II:** List of the PBPK Model Parameters [38]

Human PBPK model parameters								
Tissue	Volume, V%		Blood flow, Q%	Partition coefficient, *K*_*ptu*_			Lysosomal volume, V%	
				CQ	HCQ			
Brain	2		11.4	23	26		0.05	
Heart	0.5		4	91	102		0.1	
Kidney	0.4		17.5	261	312		0.05	
Skin	3.7		5.8	69	83		0.1	
GI	1.7		17.6	126	151		0.1	
Spleen	0.21		0.5	166	198		0.1	
Liver	2.6		4.6	237	284		0.2	
Muscle	40		19.1	81	96		0.1	
Slow	35.7		9.4	21	26		0.1	
Remaining				10	10		0.1	
Arterial	3.4							
Venous	4.0							
Respiratory model parameters								
Parameter, Symbol		Units	Upper airway	Conductional airway		Translational airway	Pulmonary alveolar	
Blood flow, *Q*		%	0.25	0.75		0.67	100	
Lysosomal volume, *V*		%	8	8		8	0.1	
Surface area, *SA*		cm^2^	138	2E3		6.22E3	540E3	
Cellular thickness, *T*		cm	1.5E-2	7.5E-3		3E-3	5E-4	
Cilia layer thickness, *T*		cm	7E-4	7E-4		7E-4	1E-5	
Mucus thickness, *T*		cm	8e-4	4E-4		2E-4		
Mucus clearance, *Kmcc*		1/min	0.08	3.2E-2		4.9E-3		
Absorption and metabolism parameters								
Drug	Oral absorption, *KA*, (1/min)	Fraction absorbed, *Fa*	Liver clearance, *CL*, (mL/min)	Kidney clearance			P-gp kinetics	
				GFR (mL/min)	Vmax (ng/mL/min)	Km (ng/mL)	Vmax (ng/cm^2^/min)	Km (ng/mL)
CQ	5E-3	1	11.1	90	515	3.2E5	9.92E5	3.68E3
HCQ	4.8E-3	0.75	12.5	90	541	3.36E5	4.2E6	3.86E3

GFR, glomerular filtration rate; P-gp, P-glycoprotein

To perform sensitivity analysis, the model parameters were varied one at a time from its nominal value while the remaining were kept constant. The maximum AUC (*AUC*_*max*_) and mean AUC (*AUC*_*mean*_) for the explored parameter was estimated to compute the sensitivity index (*SI*) using Eq. 17. The SI was normalized to the total sum of SI for all the key model parameters.

17 $$SI=\frac{AU{C}_{max}- AU{C}_{mean}}{AU{C}_{mean}}$$

## Results

### Qualification of *In Vitro* Kinetics of CQ and HCQ

**Fig. 2:**
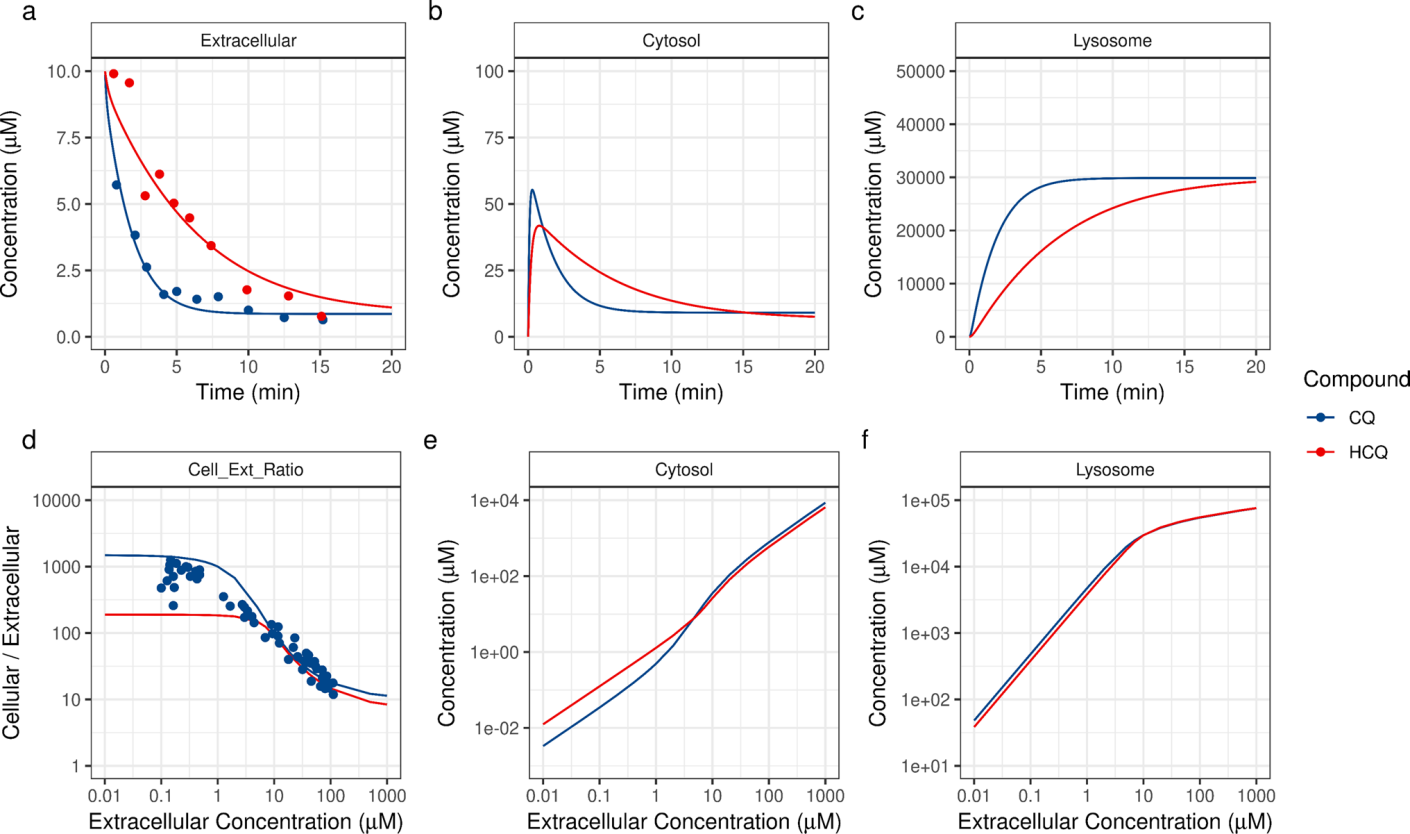
In vitro kinetics of chloroquine (CQ) and hydroxychloroquine (HCQ) in isolated rat hepatocytes. Uptake of CQ and HCQ leads to a decline in the extracellular concentrations of the compounds (a), followed by changes in their cytosolic concentrations (b) and an increase in their lysosomal concentrations (c). Model-predicted (solid line) concentration-dependent accumulation ratios (i.e., ratio of total cellular to extracellular concentrations) (d) and changes in the cytosolic (e) and lysosomal (f) concentrations after 15 min of exposure to different concentrations of CQ and HCQ. Experimental data (dots) were obtained from MacIntyre *et al*. [42, 47].

The cellular uptake kinetics of 10 μM CQ and HCQ were measured in isolated rat hepatocytes by MacIntyre *et al*. [42]. For the present study, the *in vitro* model parameters were obtained from Trapp *et al*. and MacIntyre *et al*. [25, 42]. The permeabilities of the unionized species were estimated by using initial values from Trapp *et al*. to fit the experimental data, and those of the protonated species were set at 6.5 log units lower (6.5 for +1 and 13 for +2 charge) than the permeabilities of the unionized species [39]. Despite the structural similarity of the two compounds, HCQ had a lower rate of cellular uptake than CQ, and both compounds showed similar levels of accumulation after 20 min of *in vitro* exposure (Fig. 2A). The absorption half-lives of CQ and HCQ were 1.34 and 4.56 min, respectively. The model-predicted cytosolic concentrations of CQ and HCQ reached a maximum (C_max_) at 55.2 and 41.7 μM, respectively (Fig. 2B). CQ and HCQ were predicted to be accumulated in lysosomes by reaching concentrations of 29.9 and 29.2 mM, respectively (Fig. 2C). Further, the *in vitro* model was qualified at different extracellular concentrations by comparing the model-predicted ratios of total cellular and extracellular concentrations to the experimental measurements [47]. Although an increase in the extracellular concentrations of CQ and HCQ caused a decrease in the ratio between the total cellular and extracellular concentrations (Fig. 2D), the cytosolic concentrations showed a linear increase (Fig. 2E). For both compounds, lysosomal uptake was linear at extracellular concentrations below 5 μM (Fig. 2F) and decreased at higher extracellular concentrations, as the increased lysosomal accumulation caused a decrease in the acidic pH (making the environment less acidic), thus limiting any further uptake.

**Fig. 3:**
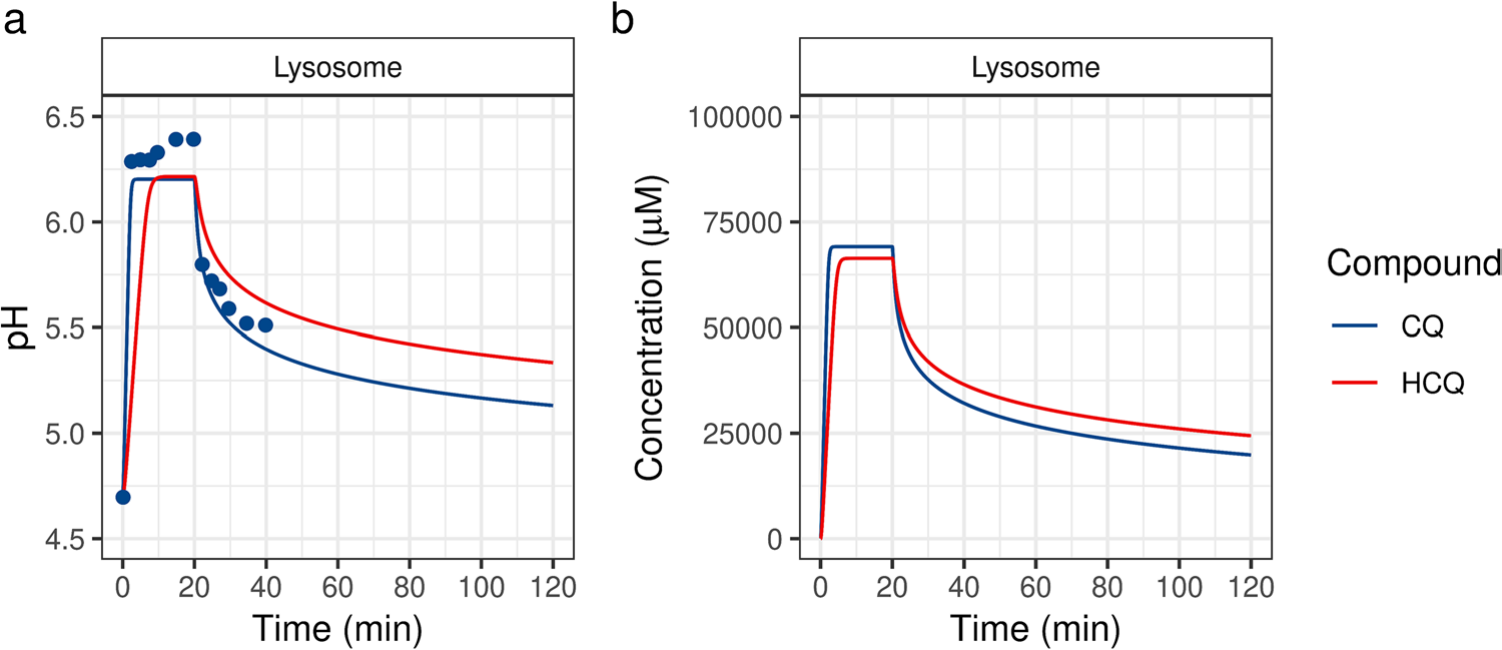
In vitro lysosomal kinetics of chloroquine (CQ) and hydroxychloroquine (HCQ) in isolated mouse peritoneal macrophages. Model-predicted (solid line) in vitro lysosomal pH (a) and lysosomal concentrations (b) after a 20-min exposure to 100 μM CQ or HCQ. Experimental data (black dots) were obtained from Ohkuma and Poole [48].

To determine the influence of CQ exposure on lysosomal pH, Ohkuma and Poole perfused isolated mouse peritoneal macrophages with 100 μM of CQ for 20 min [48]. In the present study, we simulated the *in vitro* ion-trapping model by using the same parameters, and our model-predicted lysosomal pH changes were consistent with the experimental data reported by Ohkuma and Poole [48] (Fig. 3A). Within 2.5 min of exposure, CQ reached a lysosomal concentration of 69.1 mM and caused the lysosomal pH to change from 4.7 to 6.2. (Fig. 3B). Removal of the extracellular CQ caused a decrease in the lysosomal CQ concentrations, with a terminal half-life of 351 min. Similarly, in the model-predicted lysosomal changes in response to extracellular exposure to 100 μM of HCQ, the lysosomal HCQ levels reached a maximum concentration of 66.4 μM, and removal of the extracellular HCQ led to a terminal half-life of 511 min. The rapid rise and slower decline in lysosomal concentrations were attributed to the permeabilities of the neutral and ionic species.

### Simulating CQ and HCQ Kinetics in Cell Lines and Primary Human Airway Cell Cultures

**Fig. 4:**
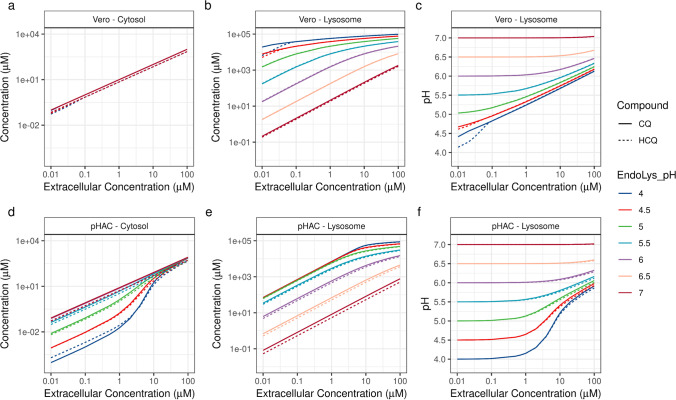
Simulated in vitro kinetics of chloroquine (CQ, solid line) and hydroxychloroquine (HCQ, dotted line) in the Vero cell line and a primary human airway cell culture (pHAC). Model-predicted changes in the cytosolic (a, d) and lysosomal (b, e) concentrations of the compounds and lysosomal pH (c, f) for different extracellular concentrations and pH levels representing early endosomes (pH 6.8–6.1), late endosomes (pH 6.0–4.8), and lysosomes (pH 4.5) [9].

Because cytosolic accumulation of CQ and HCQ may drive immunomodulatory signaling and lysosomal accumulation induce pH changes that affect endocytic-pathway-mediated viral replication, we performed simulations that mimicked the exposure of a 48-h cell line and primary human airway cell (pHAC) culture to various concentrations of CQ and HCQ at different endolysosomal pH levels (Fig. 4). As multiple cell lines are used to evaluate the efficacy of anti-viral compounds and the model framework can be adapted to any cell-based system of interest, we choose to simulate a cell line that is sensitive to lysosomotropic agents. Cell lines such as Vero (ATCC® CRL-1586™)—a monkey kidney epithelial cell line with a cell diameter of 17 μm—are usually cultured in a two-dimensional environment by completely soaking the cells in a cell culture medium containing the compound of interest. To simulate the kinetics of compound uptake, we assumed the Vero cell lysosomal volume to be 1% of its cellular volume and in the same order of magnitude as that in other cell lines [25, 49]. The extracellular CQ and HCQ concentrations in the Vero cell line simulations were set to be constant, because bulk concentration changes were assumed to be minimal as well as in order to eliminate the dependence on differences in intracellular lysosomal volume (Fig. S1). A 48-h exposure to different extracellular concentrations of CQ and HCQ led to a linear increase in the Vero cell cytosolic concentrations of the two compounds (Fig. 4A). At a low extracellular concentration of 0.01 μM and an initial lysosomal pH of 4.5, the ratio between the lysosomal and cytosolic concentrations of CQ and HCQ in the Vero cells was over 76,000, and the ratio between the lysosomal and extracellular concentrations was over 758,000 (Figs. 4A and B). This increase in lysosomal accumulation neutralized the lysosomal pH, which, in turn, decreased any further ion trapping of CQ and HCQ at high extracellular concentrations (Figs. 4B, C, E, and F).

The kinetics of HCQ in the pHAC culture was validated in our earlier study by incorporating physiologically relevant parameters [38]. We simulated basolateral exposure of CQ and HCQ in pHAC cultures to mimic oral administration. Unlike in Vero cells, CQ and HCQ show nonlinear accumulation in pHAC cytosol, which is attributed to their P-gp-mediated transport to the apical surface fluid and to the differential lysosomal volume (Fig. 4D). The literature-reported *in vitro* effective concentrations of CQ and HCQ in different cell lines infected with respiratory viruses range between 0.1–10 μM [50–55]. For an initial lysosomal pH of 4.5, the Vero cell model-predicted cytosolic concentrations, lysosomal concentrations, and lysosomal pH changes for low and high bound *in vitro* effective CQ extracellular concentrations (0.1–10 μM) were 0.99–99.1 μM, 21.1–57.1 mM, and 4.96–5.74, respectively (Figs. 4A, B, and C). The corresponding changes for HCQ were 0.7–70.2 μM, 21.1–57.5 mM, and 4.96–5.75, respectively. Achieving similar levels of cytosolic, lysosomal effective concentrations, and lysosomal pH changes in an *in vitro* pHAC culture system would require extracellular exposure to 3.26–33.7 μM of CQ and 3.37–43.4 μM of HCQ (Figs. 4D, E, and F).

### Model-Predicted Human PK for Oral and Pulmonary Administration of CQ and HCQ

**Fig. 5:**
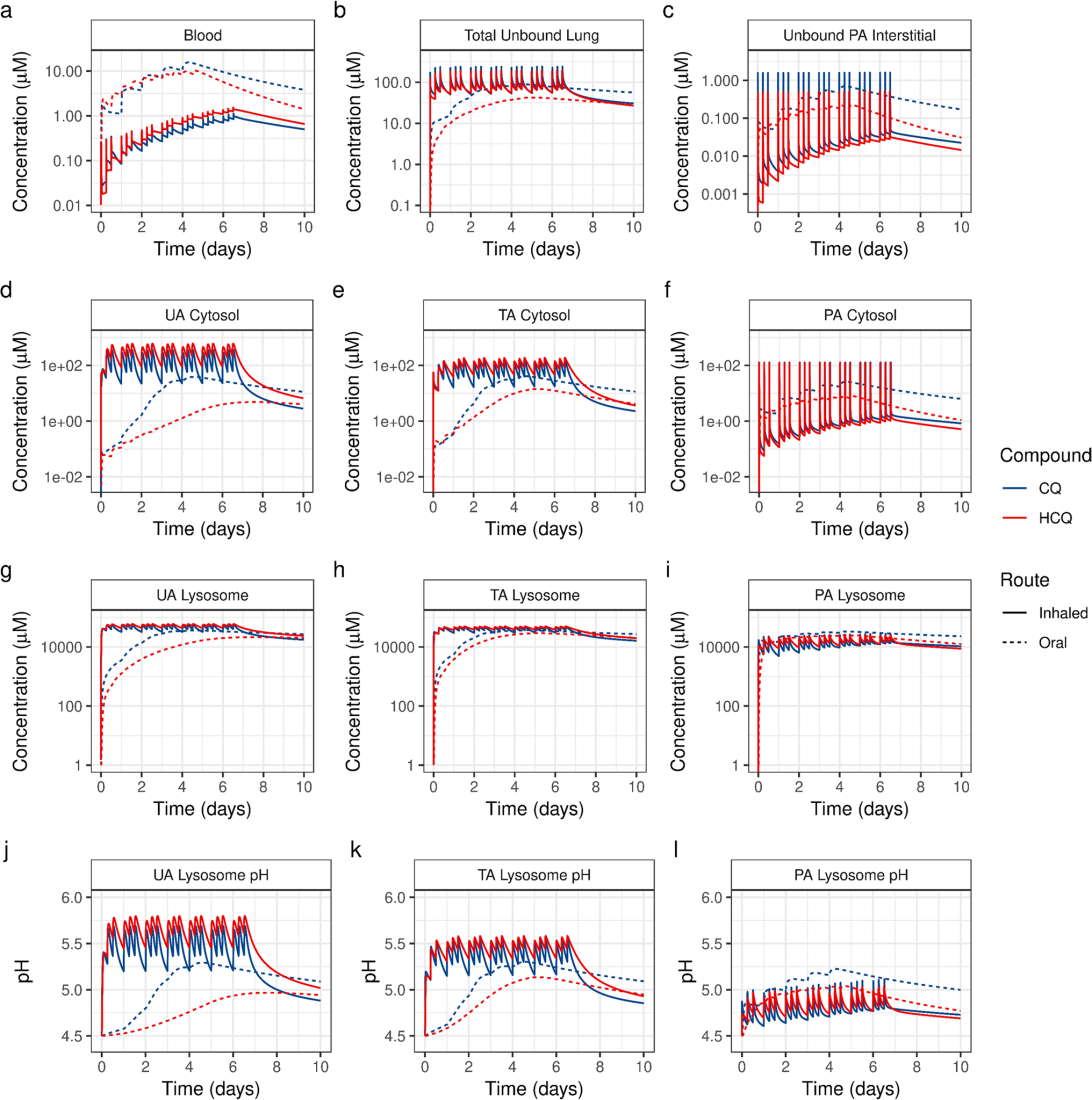
PBPK model-predicted kinetics for dosing regimens of chloroquine (CQ) and hydroxychloroquine (HCQ) in humans. The simulated inhaled dosing regimen (solid line) of 60 mg t.i.d. for CQ and HCQ, and the oral dosing regimens (dotted line) are 310 mg q.d. for 5 days for CQ [29] and 600 mg b.i.d. on day 1 and 400 mg q.d. on days 2–5 for HCQ [34]. Model-predicted changes in the (a) blood, (b) lung (total unbound drug), (c) pulmonary alveolar interstitial (unbound drug), (d) upper airway cytosolic, (e) transitional airway cytosolic, (f) pulmonary alveolar cytosolic, (g) upper airway lysosomal, (h) transitional airway lysosomal, and (i) pulmonary alveolar lysosomal concentrations of the drugs and in the (j) upper airway lysosomal pH, (k) transitional airway lysosomal pH, and (l) pulmonary alveolar lysosomal pH. UA, upper airway, TA, transitional airway; PA, pulmonary alveolar region.

The human PBPK models for CQ and HCQ were developed and qualified to experimental data in our previous study [38]. Figure 5 shows the simulated concentration profiles for oral dosing regimens of CQ (310 mg q.d. for 5 days) [29] and HCQ (600 mg b.i.d. on day 1 and 400 mg b.i.d. on days 2–5) [34]. The blood concentrations of both HCQ and CQ increased with repeated oral dosing, leading to an overall 5.6- (HCQ) to 5.8- (CQ) fold increase in total unbound lung concentrations, respectively (Figs. 5A and B). The model-predicted total lung unbound trough concentrations for oral dosing regimens of CQ and HCQ were 77.6 and 33.5 μM, respectively, on day 5 (Fig. 5B). The concentrations of unbound drugs in the pulmonary alveolar interstitium reached the low bound *in vitro* EC_50_ values (of 0.1 μM) for oral dosing of CQ and HCQ (Fig. 5C).

As the upper and bronchial airway regions are more susceptible to viral infections, we simulated the changes in cytosol concentrations, lysosomal concentrations, and lysosomal pH in different regions of the respiratory tract. The trough cytosolic concentrations in different regions of the lungs for oral dosing were less than 28.4 and 7.54 μM for CQ and HCQ, respectively (3.5 and 9.3 times lower than the high bound *in vitro* effective cytosolic concentrations, respectively) (Figs. 5D–F). Similarly, the lysosomal concentrations were 1.7 (CQ) to 2.3 (HCQ) times lower than the high bound *in vitro* effective lysosomal levels (Figs. 5G–I). The short-term oral dosing regimens that are currently used for treating VRIs did not reach the *in vitro*-predicted effective lysosomal pH of 5.74 (Figs. 5J, K, and L).

Because the airway cytosolic and lysosomal concentrations for oral dosing had increased slowly, we simulated the PK for pulmonary administration of 60 mg t.i.d. of CQ and HCQ (180 mg total daily nominal dose). Pulmonary drug delivery is complex, with significant device losses and varied regional lung deposition depending on the physicochemical properties of the aerosol and the inhalation maneuvers [56]. Hence, we assumed an overall deposition of 35% (21 mg) in the respiratory tract, with 8.75% (5.25 mg) deposited in the UA, CA, TA, and PA regions, respectively. As we aim to understand the preliminary airway dosimetry of inhaled CQ and HCQ, we assumed instant dissolution of the deposited compound in the airway mucus [38]. Unlike oral dosing, pulmonary administration delivers the maximum lung concentrations (C_max_) on day 1, while maintaining low systemic exposure (Figs. 5A and B). The model-predicted total unbound trough and maximum lung concentrations for pulmonary administration were 50.4 and 235 μM for CQ and 52 and 176 μM for HCQ, respectively (Fig. 5B). The CQ and HCQ parameters reached their high bound *in vitro* efficacious levels in terms of UA cytosolic concentrations (~100 μM), UA lysosomal concentrations (~57 mM), and UA lysosomal pH (~5.75) (Figs. 5D, G, and J). Although the CQ and HCQ levels reached their *in vitro*-predicted lower bound efficacious levels in the TA region (Figs. 5E, H, and K), it is possible to obtain higher and sustained levels with an increased regional deposited dose.

The transport of CQ and HCQ across the PA region was rapid, with unbound PA interstitial concentrations reaching maximum levels at 0.12 and 0.27 min post-inhalation, respectively (Fig. 5C). In contrast to the kinetics across the UA region, the cytosolic concentrations of CQ and HCQ in the PA region ranged between 0.1 and 120 μM owing to the rapid absorption of the two compounds, which led to a slower increase in the PA lysosomal pH (Figs. 5F, I, and L). It is possible to achieve a higher effective change in an airway region of interest by increasing the total inhaled nominal dose or modifying the aerosol physiochemical properties for increased regional deposition [38]. Given the differential kinetics of absorption and clearance of CQ and HCQ in different regions of the respiratory tract, it would require more than 1 day of inhaled dosing to maintain efficacious cytosolic and lysosomal levels in the respiratory tract, while even 3 days or more of oral dosing may not help achieve high bound *in vitro* effective concentrations in the respiratory tract (Figs. 5D–L).

### Optimal Dosing Regimens for CQ and HCQ

**Fig. 6:**
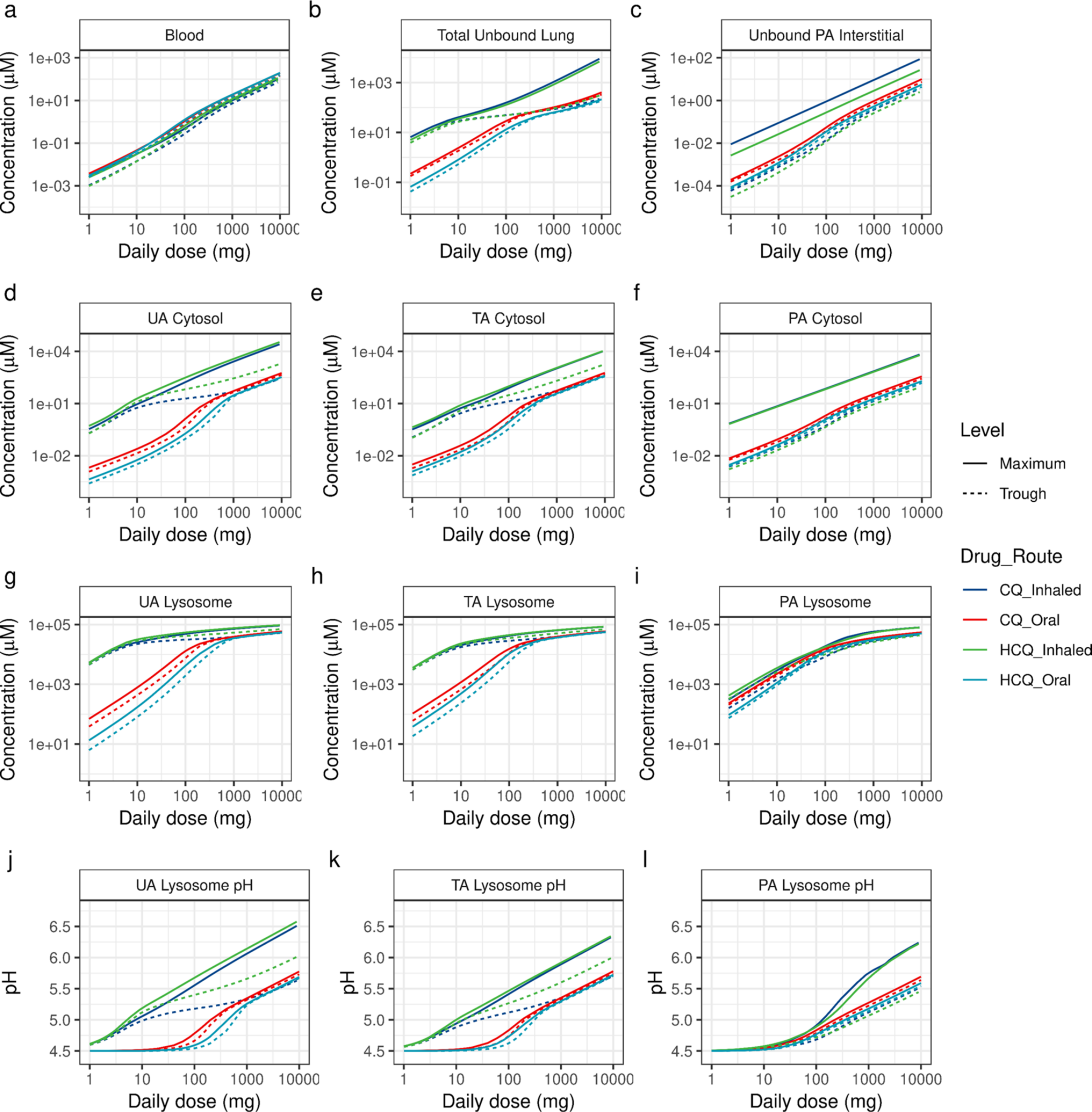
Model-predicted maximum and trough levels for various doses of chloroquine (CQ) and hydroxychloroquine (HCQ) administered by the oral and inhalation routes for 7 days. Simulated inhalation with a t.i.d. dosing regimen and oral administration with a b.i.d. dosing regimen. Trough levels (dashed line) are from day 6, and the maximum levels (solid line) are from the overall prediction. Model-predicted changes in the (a) blood, (b) lung (total unbound drug), (c) pulmonary alveolar interstitial space, (d) upper airway cytosolic, (e) transitional airway cytosolic, (f) pulmonary alveolar cytosolic, (g) upper airway lysosomal, (h) transitional airway lysosomal, and (i) pulmonary alveolar lysosomal concentrations and in the (j) upper airway lysosomal pH, (k) transitional airway lysosomal pH, and (l) pulmonary alveolar lysosomal pH. UA, upper airway, TA, transitional airway; PA, pulmonary alveolar region.

Next, we used the model to identify the optimal efficacious doses needed to obtain desired local concentrations of CQ and HCQ in the respiratory tract. For comparison, we simulated oral administration with a b.i.d. dosing regimen and pulmonary administration with a t.i.d. dosing schedule for 7 days to predict the trough levels of the compounds (Fig. 6). Considering the rapid transport kinetics of both compounds across the PA region (Fig. 5F) and the higher permeability of CQ, trough level changes alone are poor indicators for inhaled compound kinetics (Fig. 5C, F, I, and L). Therefore, we calculated other PK indices, such as the maximal levels (C_max_) for multiple oral and inhaled doses of CQ and HCQ (Fig. 6). For inhaled CQ and HCQ, the total unbound lung concentrations for inhaled CQ and HCQ were predicted to be higher than those for the oral doses while the blood and PA interstitium concentrations remained lower than those for oral dosing (Figs. 6A, B, and C). Oral administration of a total daily dose of >4000 mg, a toxic dose, could achieve high bound effective cytosolic trough concentrations of 100 μM and a lysosomal pH change to 5.48 (Figs. 6D–L). An inhaled total daily nominal dose of >150 mg CQ or > 120 mg HCQ would achieve maximum efficacious cytosolic concentrations (>107 μM for CQ and > 80 μM for HCQ) in all regions of the respiratory tract (Figs. 6D, E, and F) while providing maximum overall unbound lung concentrations of 211 μM for CQ and 146 μM for HCQ (Fig. 6B). It would require a higher inhaled nominal dose of CQ (210 mg) or HCQ (180 mg) to change the lysosomal concentrations in the UA, TA, and PA regions to obtain maximum lysosomal pH changes to 5.75, 5.58, and 5.04, respectively (Figs. 6G–L). The model-predicted UA cytosolic trough concentrations of CQ for a daily inhaled nominal dose of 240 mg was 4.26 times lower than that of HCQ (Fig. 6D) owing to compound-specific permeability differences. Therefore, a higher dose of CQ needs to be inhaled in order to achieve efficacious trough levels in the UA and TA regions (Figs. 6D, E, G, H, J, and K). A higher daily inhalation dose of CQ would also be required to obtain high bound efficacy levels in the PA region (Figs. 6F, I, and L). However, for nominal doses above 300 mg CQ, the trough concentrations for oral and inhaled administration were similar (Fig. 6B). The trough lysosomal levels in different regions of the respiratory tract are challenging to achieve and may require very high doses (Figs. 6G–L).

**Fig. 7:**
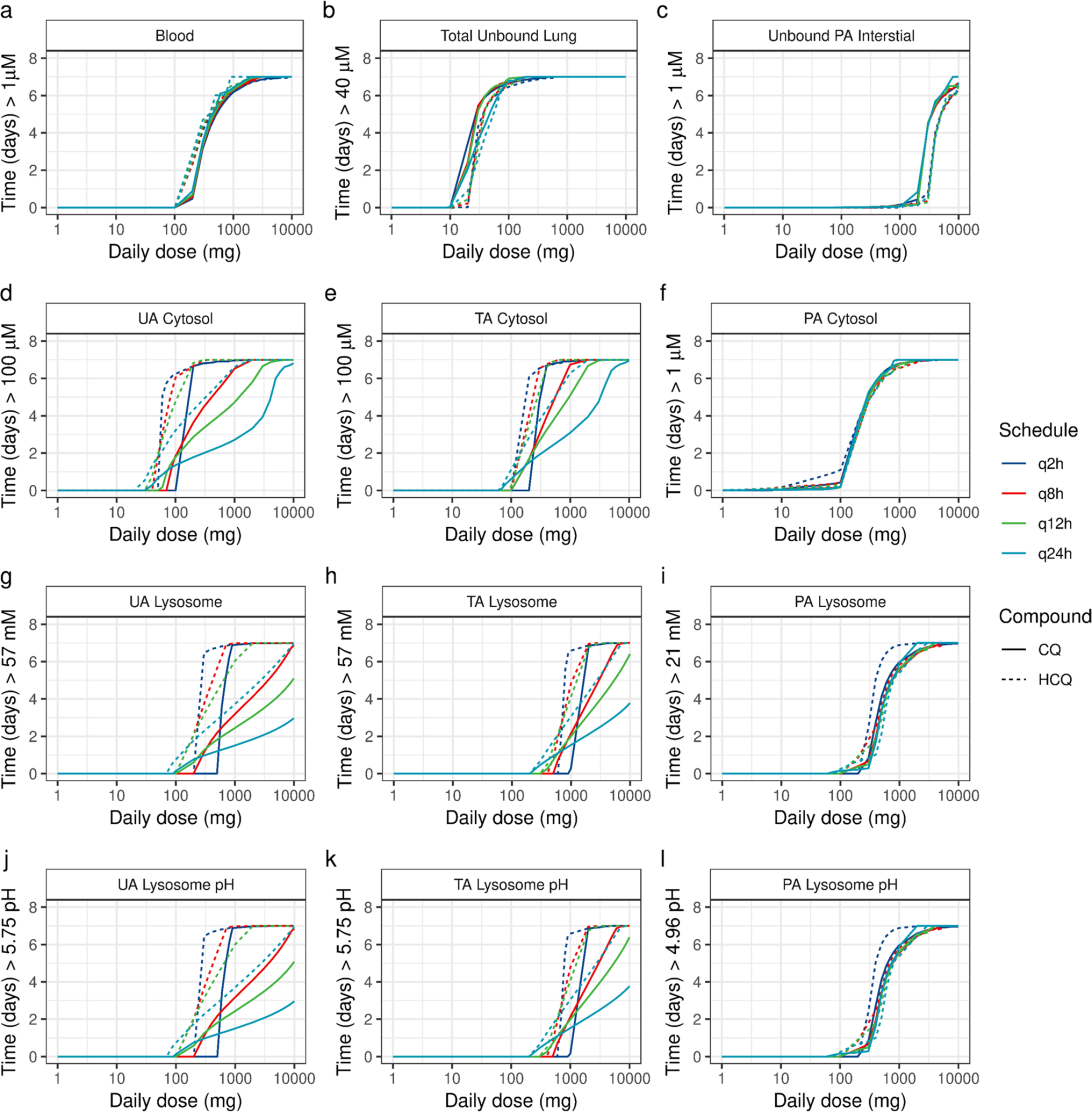
Model-predicted time above effective levels for various inhaled doses and dosing schedules of chloroquine (CQ, solid line) and hydroxychloroquine (HCQ, dotted line) in humans. Model-predicted changes in the (a) blood, (b) lung (total unbound drug), (c) pulmonary alveolar interstitial (unbound drug), (d) upper airway cytosolic, (e) transitional airway cytosolic, (f) pulmonary alveolar cytosolic, (g) upper airway lysosomal, (h) transitional airway lysosomal, and (i) pulmonary alveolar lysosomal concentrations and in the (j) upper airway lysosomal pH, (k) transitional airway lysosomal pH, and (l) pulmonary alveolar lysosomal pH. UA, upper airway, TA, transitional airway; PA, pulmonary alveolar region; q2h, dosing every 2 h; q8h, dosing every 8 h; q12h, dosing every 12 h; q24h, dosing every 24 h.

As the PK indices of CQ and HCQ for treating VRI are not established, we also simulated the influence of various dosing schedules by conserving and fractionating the total inhaled daily nominal dose to maximize the duration of exposure (Fig. 7). The q2h (dosing every 2 h), q8h (dosing every 8 h), q12h (dosing every 12 h) and q24h (dosing every 24 h) dosing schedules (Fig. 7B) could achieve a duration of total unbound lung exposure above 40 μM (time > 40 μM) for inhaled CQ and HCQ. But, depending on the administered total daily dose, these dosing schedules could lead to different exposures kinetics in the airway regions (Figs. 7D–L). Owing to its higher permeability, CQ showed different regional times above effective levels in the UA and TA regions and thus required higher doses of administration than HCQ (Figs. 7D, E, G, H, J, and K). Although the overall duration of CQ and HCQ exposure in the UA to TA regions could be maximized by fractionating high doses, low doses could be administered in a q12h or q24h dosing regimen (Figs. 7D, E, G, H, J, and K). As the regional surface area and tissue volumes increase along the respiratory tract, a higher deposited dose could maximize the duration of exposure. For example, it would require a 2-fold higher UA regional deposited dose (Figs. 7D, G, and J) to maximize the TA regional exposure (Figs. 7E, H, and K). Similarly, the total inhaled daily nominal dose required for maintaining lysosomal changes in the UA, CA and TA regions are 4-fold higher than the doses required to obtain cytosolic concentrations (Figs. 7D, E, G, H, J, and K). Although the compounds reach the C_max_ for higher effective levels in the PA region (Figs. 6F, I and L), owing to their rapid transport across the PA region, none of the dosing schedules would maximize the duration of exposure required for maintaining high bound effective levels (Figs. 5C, F, I, and L). Instead, it is possible to achieve lower bound effective levels in the PA region for treating VRIs that require a lower EC_50_.

### Sensitivity Analysis of PBPK Model Parameters

**Fig. 8:**
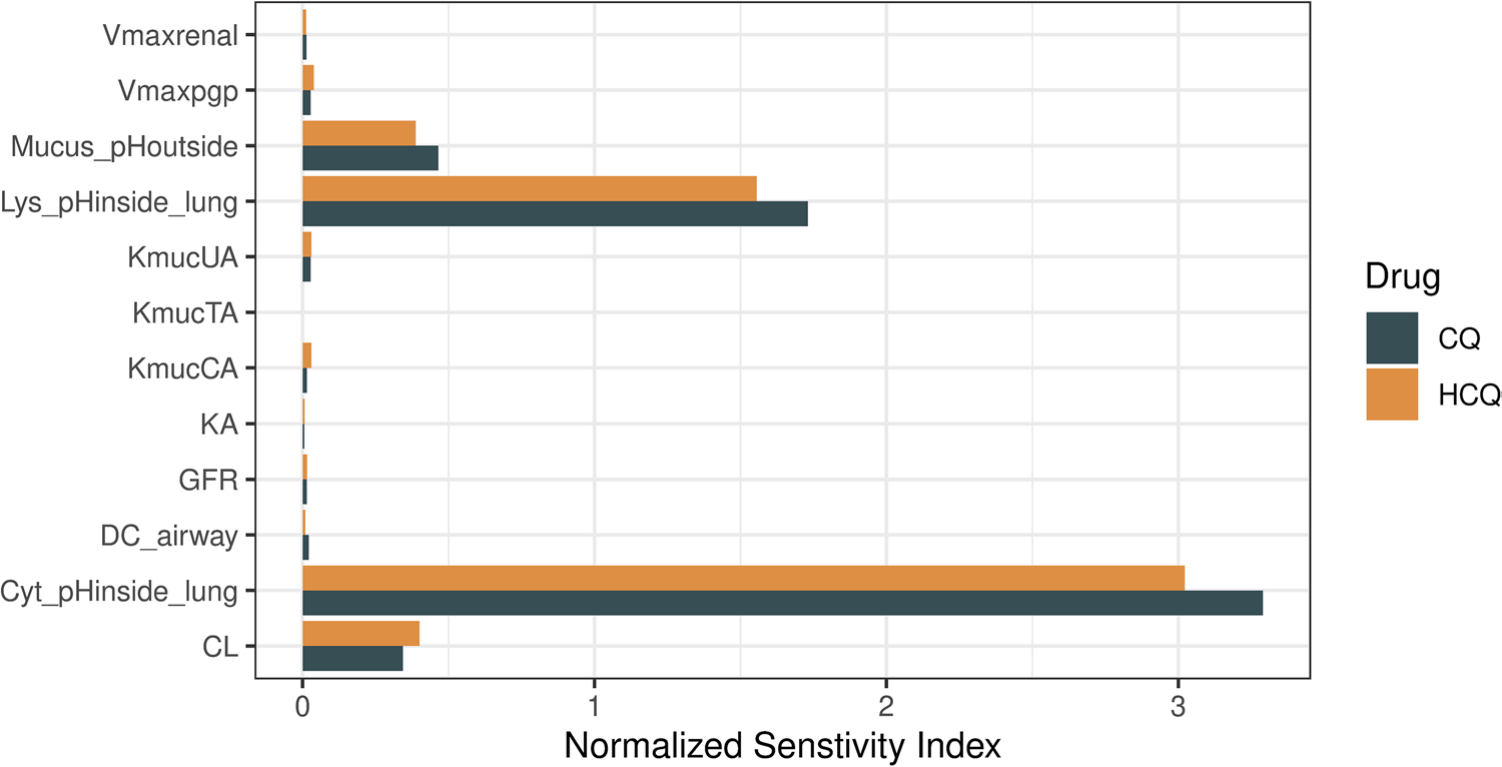
Sensitivity analysis index for the AUC of CQ and HCQ. CL, liver clearance; DC, diffusion coefficient; KA, oral absorption rate, Kmuc, mucociliary clearance rate.

As the model parameters could have a degree of uncertainty, a sensitivity analysis of key PBPK model parameters on the systemic and total lung exposure were evaluated. The PBPK model parameters were varied by 50% and simulations were performed for a pulmonary administration of 60 mg t.i.d. of CQ and HCQ. Figure 8 shows the important PBPK model parameters that vary AUC of inhaled CQ and HCQ. Interestingly, the cytosolic pH of airway tissue (Cyt_pHinside_lung) was found to be most sensitive parameter that influences both the systemic and lung concentrations. The next two sensitive parameters were epithelial lining fluid pH (Mucus_pHoutside) and intralysosomal pH in lung (Lys_pHinside_lung). As the pH is altered, the amount of neutral and ionized fractions varies in different regions contributing to differential PK. The full inhalation PK of CQ and HCQ for each varied parameter are show  in Supplemental Figs. 2–5.

## Discussion

Clinical success in VRI treatment can be achieved with an optimal route of administration and dosing schedules for obtaining efficacious local concentrations. Typically, clinical studies evaluate oral dosing regimens that deliver unbound plasma concentrations above *in vitro* EC_50_ values in cell lines. But the intracellular distribution of compounds in cell lines differs from cells lining the respiratory tract and is largely influenced by the physiochemical properties of the compound, pathophysiological state of the cells, and cell type-specific intracellular organelle volumes and active transporters. By extending the mechanistic model framework from our earlier study [38], we derived the human airway dosimetry of CQ and HCQ by linking *in vitro* model-predicted cytosolic and lysosomal changes in cell lines and pHACs to a PBPK model. The regional airway dosimetry for different dosing schedules is also further explored in the current study. We predicted that pulmonary delivery of CQ and HCQ, and not oral administration, could achieve the *in vitro*-predicted effective levels in the cytosolic and lysosomal regions along the respiratory tract.

Cell lines are widely used for evaluating the efficacy of antiviral compounds, and they produce rapid results. But the cellular characteristics of cell lines could lead to different local concentrations of compounds than those observed in primary cells (Figs. 4A and D). For example, in cells lines, the lysosomal volumes are 0.5% of the cell volume in MCF7, 0.8% in MDA-MB-231, 1.4% in T47D, and 3.7% in MDA-MB-468 cells [49]. The human respiratory tract is lined with different cell types—such as goblet cells, club cells, fibroblasts, epithelial, and macrophages—and their expression varies in different regions of the lungs. The lysosomal volumes in epithelial cells and alveolar macrophages are upto 8% of the cellular volumes [57]. Due to these differential cellular characteristics and experimental conditions, cell lines show a linear change in the cytosolic and lysosomal concentrations of CQ and HCQ, while pHACs show nonlinear intracellular accumulation (Fig. 4). As differential lysosomal volumes are one of the factors that influence the cellular retention of CQ and HCQ in the respiratory tract, prolonged exposure to CQ and HCQ could increase the size of lysosomes and modulate cell fate, and its effects remain to be further evaluated [49]. The *in vitro* EC_50_ values of CQ and HCQ in Vero cells infected with SARS-CoV-2 range between 0.72 and 6.9 μM [53, 54]. Using these *in vitro* EC50 values from cell lines could result in lack of antiviral efficacy in pHACs (Fig. 4). In pHACs, 10 μM HCQ resulted in a 2.4-fold reduction in SARS-CoV-2 nucleoprotein gene copy numbers in a previous study [58] and an exposure to 20–40 μM HCQ has been reported to decrease the SARS-CoV-2 viral load [59]. In another study, 50 μM of HCQ decreased the replication of a rhinovirus in human tracheal epithelial cells grown at the air–liquid interface by 32-fold [60]. The activation of signaling pathways for immunomodulatory effects requires that threshold concentrations of CQ and HCQ are achieved in different intracellular compartments, such as the cytosol. An exposure to higher concentrations of the compounds is necessary for achieving cytosolic threshold concentrations and the desired intracellular distribution for activating immunomodulatory effects in pHACs. Primary human epithelial cells infected with a rhinovirus generate pro-inflammatory CXC chemokines, IP-10 and RANTES, and treatment with 50 μM HCQ inhibited it [60]. Our *in vitro* kinetic modeling of HCQ in pHACs also suggests that an extracellular exposure of >43.4 μM HCQ is required to achieve efficacious cytosolic and lysosomal concentrations of the compound (Fig. 4). Our mechanistic modeling of the *in vitro* kinetics of CQ and HCQ provides the rationale for the higher effective concentrations of the two compounds in pHACs. Thus, a direct extrapolation to *in vivo* doses on the basis of cell line EC_50_ values might not result in clinical efficacy.

Both CQ and HCQ have been shown to be effective under pre- and post-viral exposure conditions, with HCQ being slightly more potent [53]. The EC_50_ values of the compunds for initiating virus-mediated immune response are lower than those required for inhibiting viral replication in different cell lines [22]. However, the *in vitro* dose–response for different VRIs needs to be intrepreted carefully, as the pathogenesis of a VRI and the fractional contribution of each mechanism (e.g., endolysosomal pH changes and immunomodulatory effects) towards clinical efficacy are not known. CQ and HCQ have been shown to be more effective in cells with an endocytic pathway as the major pathway for virus entry [53]. Both drugs can also inhibit viruses that tend to egress the cell via the Golgi–endocytic pathway post-viral replication [61]. The kinetics of endocytosis along the upper and lower respiratory tract could potentially be different and needs to be evaluated. While nasal epithelial cells express increased levels of various endocytic markers, indicating the existence of multiple mechanisms, pneumocytes have a restricted expression profile of key endocytic proteins [62]. Further analysis of the temporal dynamics in lysosomal changes and immunomodulatory signaling pathways in relevant cell systems could help understand the contribution of different mechanisms towards CQ and HCQ efficacy against VRIs.

Although the decision about selecting an optimal dosing regimen needs to be evaluated in a clinical setting by considering both the efficacy and safety profiles of the compounds, an early identification of the pharmacokinetic drivers of efficacy will enable better design of optimal dosing regimens. As an increase in duration of exposure lowered *in vitro* EC_50_ values [53], the time above the *in vitro*-predicted cytosolic and lysosomal concentrations or pH could serve as a preliminary pharmacokinetic driver of efficacy. Several dosing regimens have been simulated to identify regimens that maximize the time above effective levels. Our model predictions for the oral dosing regimens of CQ and HCQ are in agreement with previously reported tissue concentrations, which allows us to derive insights for bridging the *in vitro* and *in vivo* kinetics of the compounds for efficacy [37, 53, 63]. According to our model predictions, neither oral nor inhaled doses calculated on the basis of lung volume might achieve efficacious local concentrations in the lungs [53, 59, 64–67]. Furthermore, during lung infection, CQ and HCQ accumulation in lung tissues is predicted to increase substantially because of the pH changes in the epithelial lining fluid (ELF) [63]. But the changes in the cytosolic and lysosomal levels of accumulation for both drugs could be minimal, as the compounds may get ion-trapped in the acidic ELF; this possibility remains to be evaluated. Even if the cytosolic pH becomes acidic and increases the trapped concentrations of the drugs, the amount of unbound neutral fractions could be low. Sensitivity analysis also suggested lung pH to be one the major parameter influencing lung exposure. Hence, further research to understand the ion-balance across airway epithelia in normal and virus infected conditions could be beneficial.

Several clinical trials have evaluated various oral dosing schedules of CQ and HCQ for treating VRIs [29, 33–36, 68–70] and showed no efficacy, probabaly because they failed to achieve high bound cytosolic and lysosomal concentrations of the drugs. Furthermore, the clinical failure of CQ and HCQ during later stages of disease progression could be related to the high viral load in the PA region, which necessitates high cytosolic concentrations for immunomodulatory effects and significant changes in endolysosomal concentrations for inhibition of viral replication, which are difficult to achieve [34, 35]. In contrast, HCQ has been associated with improved patient outcomes in subjects who are diagnosed early, have low viral loads, and have received early treatment [68, 70]. An oral daily dose of 400 mg HCQ has been reported to decrease the systemic levels of a pro-inflammatory cytokine (IL-6) in subjects with a VRI [71]. Low concentrations of CQ and HCQ have been reported to inhibit nucleic acid sensors, including TLR 9 and cyclic GMP-AMP synthase, to exhibit immunomodulatory effects [22]. Hence, CQ and HCQ could show *in vivo* efficacy against VRIs with early pulmonary administration to achieve high bound cytosolic and lysosomal concentrations in the respiratory tract. An inhaled nominal dose of up to 50 mg HCQ sulphate has been found to be safe in a phase-I clinical study [72]. A clinical study has been proposed for evaluating the efficacy of a daily dose of 300 mg CQ phosphate (150 mg with a q12h dosing regimen) administered via the pulmonary route against SARS-CoV-2 (registration number: ChiCTR2000029975). Even though our PBPK model predicted that oral and pulmonary administration of high nominal doses of CQ and HCQ would achieve similar unbound lung trough concentrations (Fig. 6B), pulmonary administration could be beneficial, as it will provide a higher maximum concentration (C_max_) and time above effective levels with overall low systemic exposure (Fig. 6). However, the safety of pulmonary delivery of CQ and HCQ needs to be further evaluated, as the toxicological impact of such high-exposure-mediated cytosolic and lysosomal changes in the human airways is unknown.

In addition to our previous study [38], the mechanistic models presented in the current study provides a basic understanding of airway dosimetry of compounds independent of the inhalation device and formulation. Employing such translation modeling approaches during early phases of drug discovery will enable selection of compounds, perform feasibility analysis for choosing route of administration, identification of formulation requirements and selection of inhalation devices for further development. Future model development could include inclusion of formulation and device specific characteristics such as aerosol physicochemical properties, particle density and dissolution to predict PK.

## Conclusion

*In vitro* to *in vivo* translation of the efficacy of drugs for predicting optimal dosing regimens and airway dosimetry is complex. The *in vitro* effective concentrations of CQ and HCQ in pHACs are higher than those in cell lines, and, therefore, the *in vivo* doses of the compounds must be cautiously derived. Our model-predicted local concentrations suggest that oral dosing regimens of CQ and HCQ would likely not be effective against VRIs that require higher effective concentrations. Pulmonary delivery of CQ and HCQ could be effective during the early phase of VRIs, and this possibility remains to be clinically evaluated. Our translational model provides a mechanistic approach for predicting human airway PK by connecting the *in vitro* kinetics of lysosomotropic agents.

### Acknowledgments and Disclosures

We thank Anatoly Mazurov for reviewing the manuscript and providing feedback. We acknowledge Arkadiusz Kuczaj for the discussions. All authors are employees of Philip Morris International. A.R.K., F.C.M., and J.H. have patent applications pending on pharmaceutical compositions that include chloroquine or hydroxychloroquine and the uses thereof.

## Supplementary Information

ESM 1 (PDF 889 kb)
