# Supplementary material for: Translational Modeling of Chloroquine and Hydroxychloroquine Dosimetry in Human Airways for Treating Viral Respiratory Infections
Source: Pharm Res. 2022 Jan 9;39(1):57–73. doi: 10.1007/s11095-021-03152-3 (PMC8742698; doi:10.1007/s11095-021-03152-3)
Supplement: Supplementary file 1 — (PDF 889 kb) [file 11095_2021_3152_MOESM1_ESM.pdf]

Supplementary Information

Translational Modeling of Chloroquine and Hydroxychloroquine Dosimetry in Human Airways for Treating Viral Respiratory Infections

Aditya R. Kolli, Florian Calvino-Martin, Julia Hoeng

PMI R&D, Philip Morris Products S.A., Quai Jeanrenaud 5, CH-2000 Neuchâtel, Switzerland

Corresponding author:

Aditya R. Kolli
PMI R&D, Philip Morris Products S.A.
Quai Jeanrenaud 5, CH-2000 Neuchâtel, Switzerland
Tel: +41 (58) 242-2837
Email: AdityaReddy.Kolli@pmi.com

Supplemental Figures

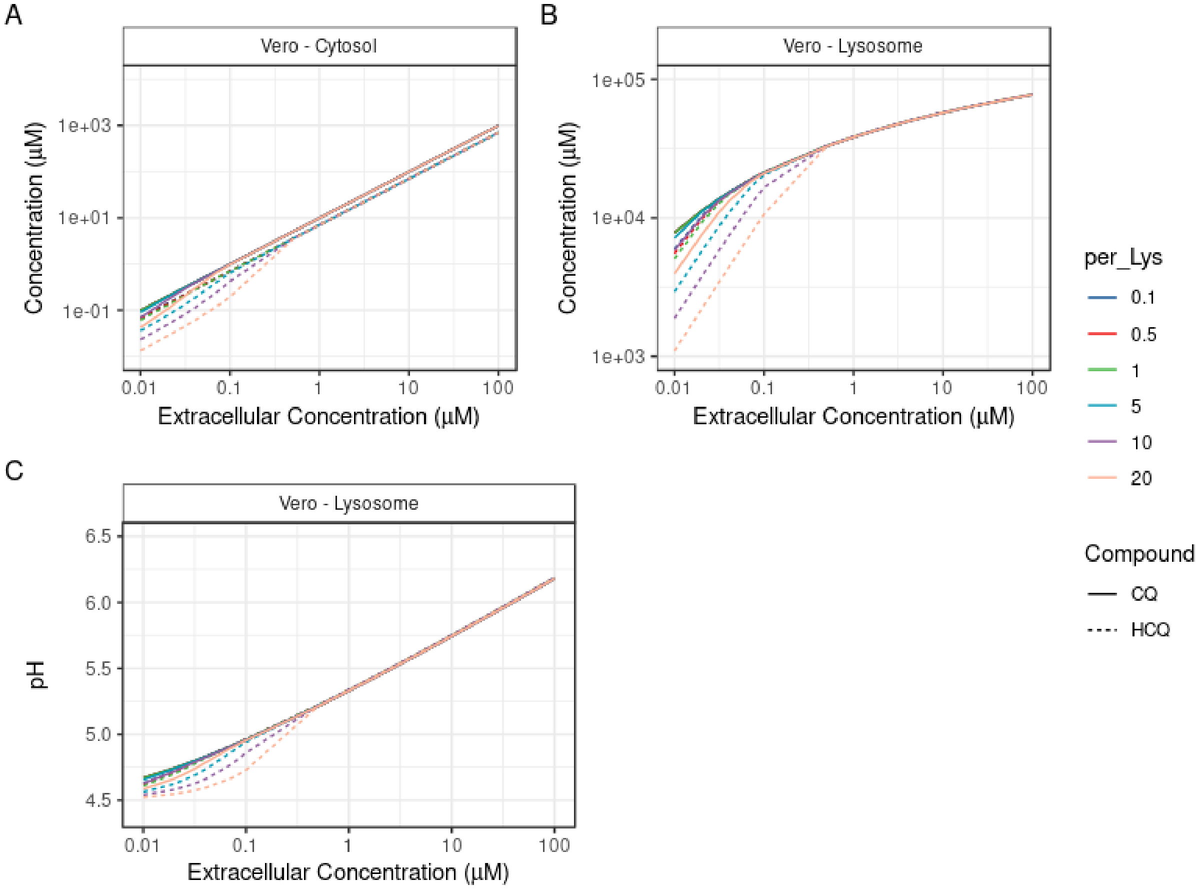

Figure S1: Influence of different lysosomal volumes (based on cellular volume) on changes in the cytosolic and lysosomal levels of CQ and HCQ at 48 h post-exposure to different extracellular concentrations of the drugs. The initial lysosomal pH was 4.5. per_Lys, percent lysosomal volume.

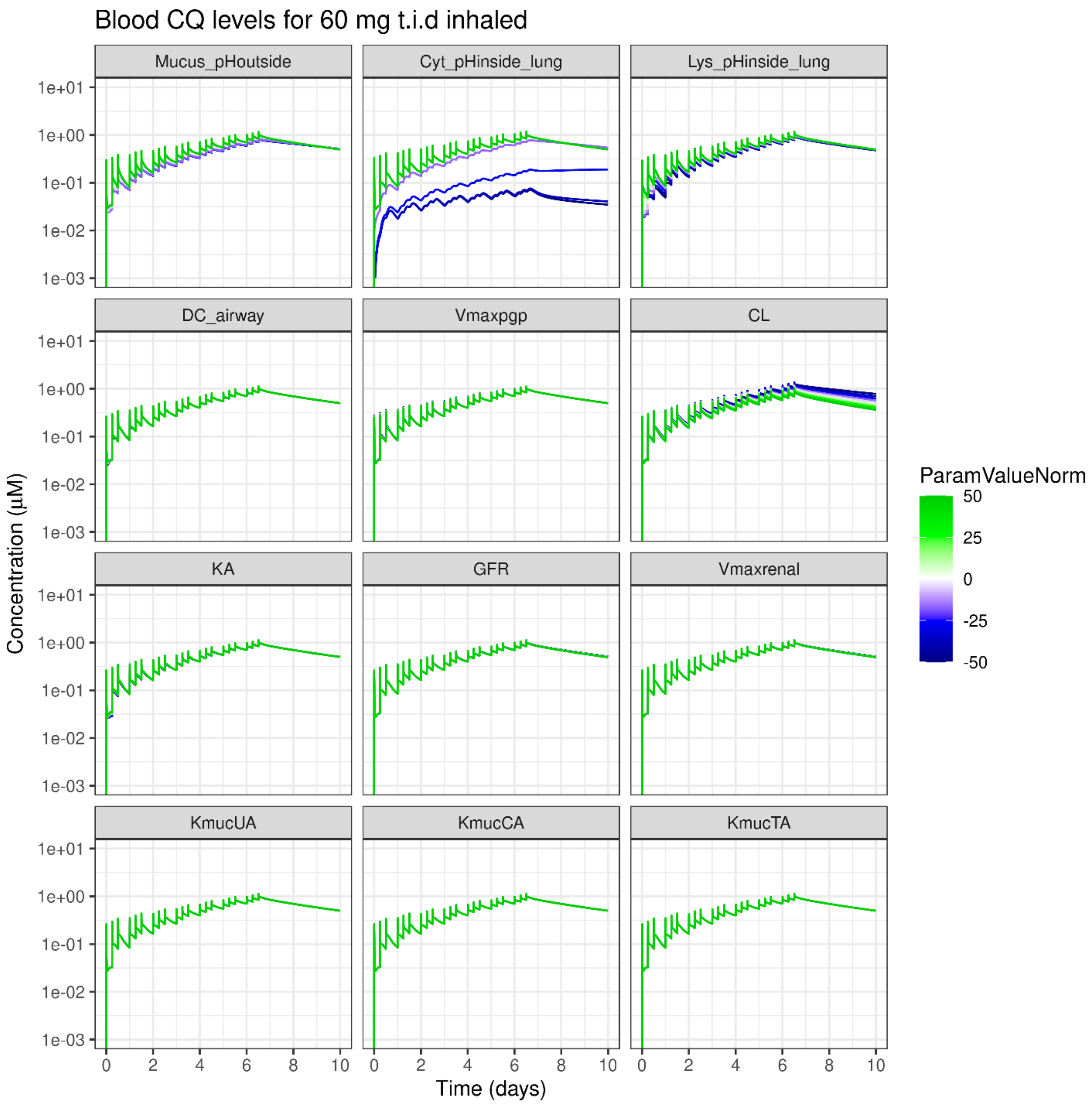

Figure S2: Influence of PBPK model parameters on systemic concentrations for 60 mg of inhaled CQ with a t.i.d dosing regimen. ParamValueNorm, is the percent change in PBPK model parameter from the nominal value.

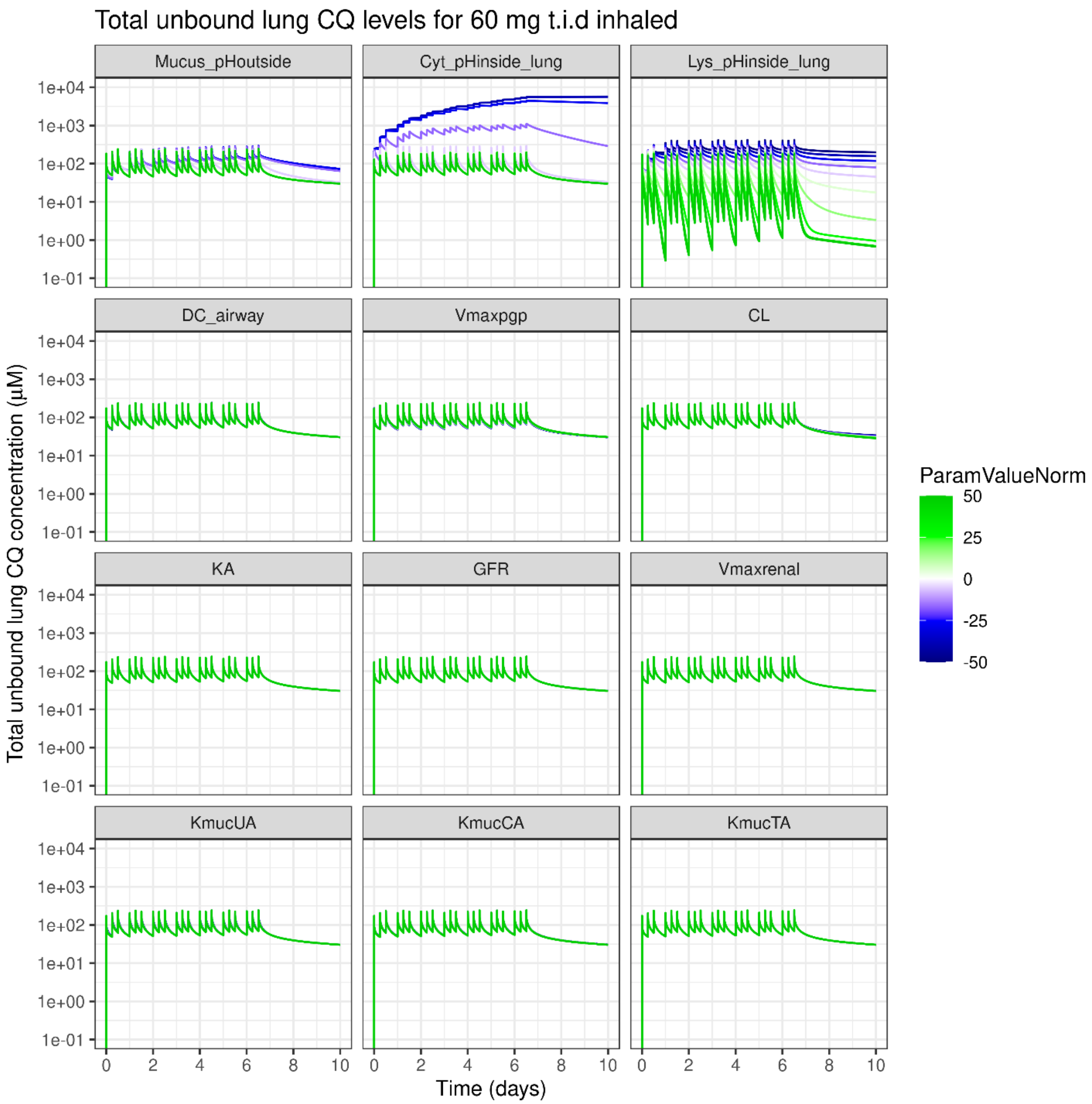

Figure S3: Influence of PBPK model parameters on total unbound lung concentrations for 60 mg of inhaled CQ with a t.i.d dosing regimen. ParamValueNorm, is the percent change in PBPK model parameter from the nominal value.

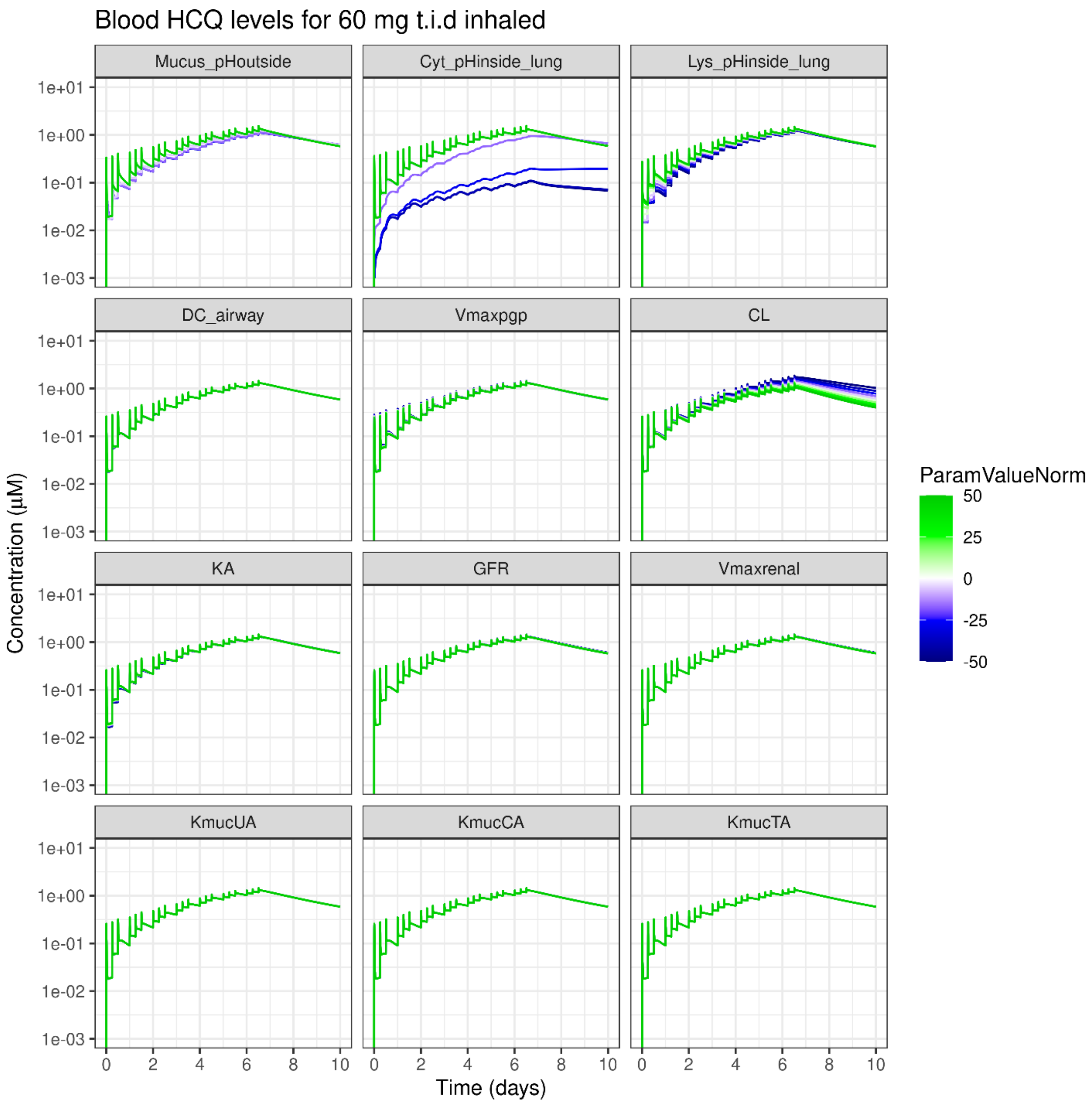

Figure S4: Influence of PBPK model parameters on systemic concentrations for 60 mg of inhaled HCQ with a t.i.d dosing regimen. ParamValueNorm, is the percent change in PBPK model parameter from the nominal value.

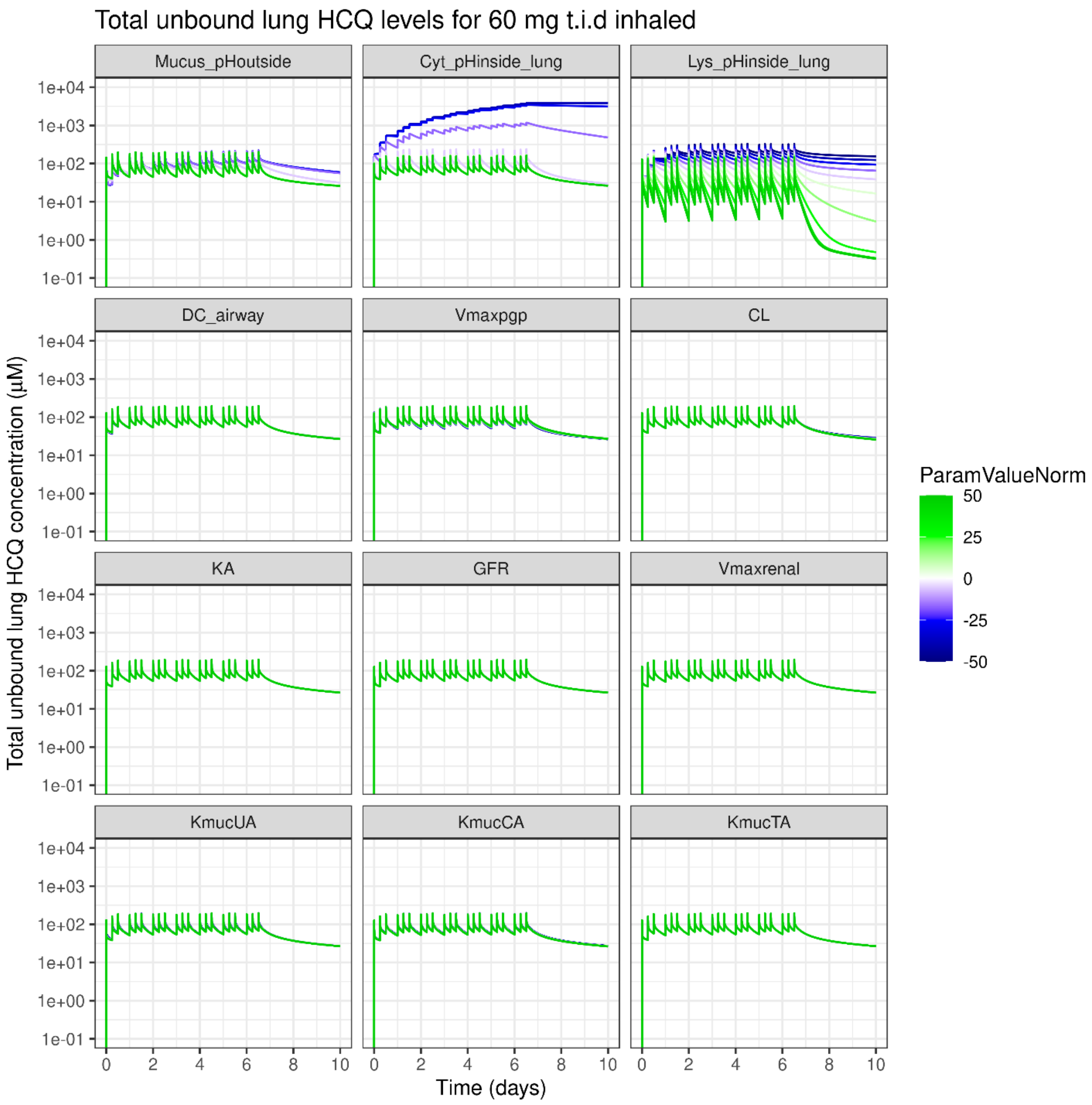

Figure S5: Influence of PBPK model parameters on total unbound lung concentrations for 60 mg of inhaled HCQ with a t.i.d dosing regimen. ParamValueNorm, is the percent change in PBPK model parameter from the nominal value.

Model Code

```
#==============================================================================#
                         Model for Isolated Rat Hepatocytes
#==============================================================================#

code <- '
$PROB
Author          : ARK
Affiliation     : PMI R&D
Date            : May 2020
Description     : Isolated Rat Hepatocyte Model

$SET
delta= 0, atol= 0, rtol=0, maxsteps=0,

$PARAM
MW = 0, logKow = 0,
pKa1 = 0, valency1 = 1, i1 = 1,
pKa2 = 0, valency2 = 2, i2 = 1,
Faraday = 0,
R = 0,
Temp = 0,
pn = 0,
pnz = 0,
Vol_Ext = 0,
Pkd_Volume = 0,
Lys_fraction = 0,
Cyt_Diameter = 0,
Cyt_pHinside = 0,
Cyt_pHoutside = 0,
Cyt_lipid= 0,
Cyt_water = 0,
Cyt_Ionstrength = 0,
Cyt_E = 0,
Lys_Diameter = 0,
Lys_pHinside = 0,
Lys_pHoutside = 0,
Lys_lipid = 0,
Lys_water=0,
Lys_Ionstrength = 0,
Lys_E = 0,
Buff_cap = 0;
gamman = 0;
gammad1 = 0;
gammad2 = 0;

$MAIN
double Vcytosol  = (4/3) * 3.14 * pow(Cyt_Diameter/2, 3);
double Vlysosome = (4/3) * 3.14 * pow(Lys_Diameter/2, 3);
```

```
double Vol_Cyt = (Pkd_Volume/100) * Vol_Ext;
double Vol_Lys = (Lys_fraction/100) * Vol_Cyt;
double SA_cell = Vol_Cyt/Vcytosol * 4*3.14*pow(Cyt_Diameter/2, 2);
double SA_lys = Vol_Lys/Vlysosome * 4*3.14*pow(Lys_Diameter/2, 2);
double Kow = pow(10, logKow);
double logKowdiss = logKow - (fabs(valency1)) *6.5;
double Kowdiss = pow(10, logKowdiss);
double logKowdiss2 = logKow - (fabs(valency2))*6.5;
double Kowdiss2 = pow(10, logKowdiss2);
double Pn = pow(10, logKow - pn);
double Pd1 = pow(10, logKowdiss - pn);
double Pd2 = pow(10, logKowdiss2 - pn);
// Fractions Outside
double fnout  = 1/(1+ pow(10, pKa1-Cyt_pHoutside) + pow(10, (pKa1-Cyt_pHoutside)+(pKa2-
Cyt_pHoutside)));
double fd1out = fnout * pow(10, (pKa1-Cyt_pHoutside)+(pKa2-Cyt_pHoutside));
double fd2out = fnout * pow(10, pKa1-Cyt_pHoutside);
double D1out = fd1out/fnout;
double D2out = fd2out/fnout;
//Fractions Cytosol
double Kncyt  = 1.22 * Cyt_lipid * Kow;
double Kd1cyt = 1.22 * Cyt_lipid * Kowdiss;
double Kd2cyt = 1.22 * Cyt_lipid * Kowdiss2;
double ancyt  = 1/(1+ pow(10, pKa1-Cyt_pHinside) + pow(10, (pKa1-Cyt_pHinside)+(pKa2-
Cyt_pHinside)));
double ad2cyt = ancyt * pow(10, (pKa1-Cyt_pHinside)+(pKa2-Cyt_pHinside));
double ad1cyt = ancyt * pow(10, pKa1-Cyt_pHinside);
double D1cyt = ad1cyt/ancyt;
double D2cyt = ad2cyt/ancyt;
double fncyt  = 1/(Cyt_water/gamman + Kncyt/gamman + D2cyt*Cyt_water/gammad2 +
D2cyt*Kd2cyt/gammad2 + D1cyt*Cyt_water/gammad1 + D1cyt*Kd1cyt/gammad1);
double fd1cyt =  fncyt * D1cyt;
double fd2cyt =  fncyt * D2cyt;
double Nd1cyt = valency1*Cyt_E*Faraday/(R*Temp);
double Nd2cyt = valency2*Cyt_E*Faraday/(R*Temp);
double Joutcyt = fnout*Pn + fd2out*Pd2*Nd2cyt/(exp(Nd2cyt)-1) + fd1out*Pd1*Nd1cyt/(exp(Nd1cyt)-
1);
double Jcytout = fncyt*Pn + fd1cyt*Pd1*exp(Nd1cyt)*Nd1cyt/(exp(Nd1cyt)-1) +
fd2cyt*Pd2*exp(Nd2cyt)*Nd2cyt/(exp(Nd2cyt)-1);

//Fractions Lyosome
double Knlys  = 1.22 * Lys_lipid * Kow;
double Kd1lys = 1.22 * Lys_lipid * Kowdiss;
double Kd2lys = 1.22 * Lys_lipid * Kowdiss2;
double anlys  = 1/(1+ pow(10, pKa1-Lys_pH) + pow(10, (pKa1-Lys_pH)+(pKa2-Lys_pH)));
double ad2lys = anlys * pow(10, (pKa1-Lys_pH)+(pKa2-Lys_pH));
double ad1lys = anlys * pow(10, pKa1-Lys_pH);
double D1lys  = ad1lys/anlys;
double D2lys  = ad2lys/anlys;
double fnlys  = 1/(Lys_water/gamman + Knlys/gamman + D2lys*Lys_water/gammad2 +
D2lys*Kd2lys/gammad2 + D1lys*Lys_water/gammad1 + D1lys*Kd1lys/gammad1);
```

```
double fd1lys = fnlys * D1lys;
double fd2lys = fnlys * D2lys;
double Nd1lys = valency1*Lys_E*Faraday/(R*Temp);
double Nd2lys = valency2*Lys_E*Faraday/(R*Temp);
double Jcytlys = fncyt*Pn + fd2cyt*Pd2*Nd2lys/(exp(Nd2lys)-1) + fd1cyt*Pd1*Nd1lys/(exp(Nd1lys)-1);
double Jlyscyt = fnlys*Pn + fd1lys*Pd1*exp(Nd1lys)*Nd1lys/(exp(Nd1lys)-1) +
fd2lys*Pd2*exp(Nd2lys)*Nd2lys/(exp(Nd2lys)-1);

$CMT
Out Cyto Lyso

$ODE
dxdt_Out = 1/Vol_Ext * (-SA_cell * (Joutcyt*Out - Jcytout*Cyto));
dxdt_Cyto = 1/(Vol_Cyt-Vol_Lys) * (SA_cell * (Joutcyt*Out - Jcytout*Cyto) - SA_lys * (Jcytlys*Cyto -
Jlyscyt*Lyso));
dxdt_Lyso = 1/Vol_Lys * (SA_lys * (Jcytlys*Cyto - Jlyscyt*Lyso));
double Lys_pH = Lys_pHinside + (Lyso/Buff_cap);
'
#=====================================================================#
                                   # END #
#=====================================================================#

#=====================================================================#
                     Model for cell lines – Vero cell
#=====================================================================#
code <- '
Author        : ARK
Affiliation   : PMI R&D
Date          : May 2020
Description   : Vero cell model

$SET
delta= 0, atol= 0, rtol=0, maxsteps=0,

$PARAM
MW = 0,
logKow= 0,
pKa1    = 0, valency1 = 1, i1 =   1,
pKa2    = 0, valency2 = 2, i2 = 1,
Faraday = 0,
R = 0,
Temp = 0,
Cyt_Diameter = 0,
Cyt_pHinside = 0,
Cyt_pHoutside          = 0,
Cyt_lipid              = 0,
Cyt_water              = 0,
Cyt_Ionstrength        = 0,
Cyt_E                  = 0,
Lys_Diameter           = 0,
percLys_Volume         = 0,
```

```
Lys_pHinside            = 0,
Lys_pHoutside           = 0,
Lys_lipid               = 0,
Lys_water               = 0,
Lys_Ionstrength         = 0,
Lys_E                   = 0,
Buff_cap                = 0;
pn                      = 0,
gamman                  = 0;
gammad1                 = 0;
gammad2                 = 0;


$MAIN
double Cyt_area  = 4 * 3.14 * pow(Cyt_Diameter/2, 2);
double Cyt_Volume  = 4/3 * 3.14 * pow(Cyt_Diameter/2, 3);
double Lys_Volume1  = 4/3 * 3.14 * pow(Lys_Diameter/2, 3);
double Lys_Volume  = Cyt_Volume * percLys_Volume/100;
double Lys_area  = Lys_Volume* (4)*3.14 * pow(Lys_Diameter/2, 2) / Lys_Volume1;
double Kow = pow(10, logKow);
double logKowdiss = logKow - (fabs(valency1))*6.5;
double Kowdiss = pow(10, logKowdiss);
double logKowdiss2 = logKow - (fabs(valency2))*6.5;
double Kowdiss2 = pow(10, logKowdiss2);
double Pn  = pow(10, logKow - pn);
double Pd1 = pow(10, logKowdiss - pn);
double Pd2 = pow(10, logKowdiss2 - pn);
double fnout  = 1/(1+ pow(10, pKa1-Cyt_pHoutside) + pow(10, (pKa1-Cyt_pHoutside)+(pKa2-
Cyt_pHoutside))) ;
double fd1out = fnout * pow(10, (pKa1-Cyt_pHoutside)+(pKa2-Cyt_pHoutside));
double fd2out = fnout * pow(10, pKa1-Cyt_pHoutside);
double D1out = fd1out/fnout;
double D2out = fd2out/fnout;
double Kncyt  = 1.22 * Cyt_lipid * Kow;
double Kd1cyt = 1.22 * Cyt_lipid * Kowdiss;
double Kd2cyt = 1.22 * Cyt_lipid * Kowdiss2;
double ancyt  = 1/(1+ pow(10, pKa1-Cyt_pHinside) + pow(10, (pKa1-Cyt_pHinside)+(pKa2-
Cyt_pHinside)));
double ad2cyt = ancyt * pow(10, (pKa1-Cyt_pHinside)+(pKa2-Cyt_pHinside));
double ad1cyt = ancyt * pow(10, pKa1-Cyt_pHinside);
double D1cyt = ad1cyt/ancyt;
double D2cyt = ad2cyt/ancyt;
double fncyt  = 1/(Cyt_water/gamman + Kncyt/gamman + D2cyt*Cyt_water/gammad2 +
D2cyt*Kd2cyt/gammad2 + D1cyt*Cyt_water/gammad1 + D1cyt*Kd1cyt/gammad1);
double fd1cyt =  fncyt * D1cyt;
double fd2cyt =  fncyt * D2cyt;
double Nd1cyt = valency1*Cyt_E*Faraday/(R*Temp);
double Nd2cyt = valency2*Cyt_E*Faraday/(R*Temp);
double Joutcyt = fnout*Pn + fd2out*Pd2*Nd2cyt/(exp(Nd2cyt)-1) + fd1out*Pd1*Nd1cyt/(exp(Nd1cyt)-
1);
```

```
double Jcytout = fncyt*Pn + fd1cyt*Pd1*exp(Nd1cyt)*Nd1cyt/(exp(Nd1cyt)-1) +
fd2cyt*Pd2*exp(Nd2cyt)*Nd2cyt/(exp(Nd2cyt)-1);
double Knlys  = 1.22 * Lys_lipid * Kow;
double Kd1lys = 1.22 * Lys_lipid * Kowdiss;
double Kd2lys = 1.22 * Lys_lipid * Kowdiss2;
double anlys  = 1/(1+ pow(10, pKa1-Lys_pH) + pow(10, (pKa1-Lys_pH)+(pKa2-Lys_pH)));
double ad2lys = anlys * pow(10, (pKa1-Lys_pH)+(pKa2-Lys_pH));
double ad1lys = anlys * pow(10, pKa1-Lys_pH);
double D1lys = ad1lys/anlys;
double D2lys = ad2lys/anlys;
double fnlys  = 1/(Lys_water/gamman + Knlys/gamman + D2lys*Lys_water/gammad2 +
D2lys*Kd2lys/gammad2 + D1lys*Lys_water/gammad1 + D1lys*Kd1lys/gammad1);
double fd1lys =  fnlys * D1lys;
double fd2lys =  fnlys * D2lys;
double Nd1lys = valency1*Lys_E*Faraday/(R*Temp);
double Nd2lys = valency2*Lys_E*Faraday/(R*Temp);
double Jcytlys = fncyt*Pn + fd2cyt*Pd2*Nd2lys/(exp(Nd2lys)-1) + fd1cyt*Pd1*Nd1lys/(exp(Nd1lys)-1);
double Jlyscyt = fnlys*Pn + fd1lys*Pd1*exp(Nd1lys)*Nd1lys/(exp(Nd1lys)-1) +
fd2lys*Pd2*exp(Nd2lys)*Nd2lys/(exp(Nd2lys)-1);

$CMT
Out Cyto Lyso

$ODE
dxdt_Out = 0; // no change
dxdt_Cyto = 1/(Cyt_Volume- Lys_Volume) * (Cyt_area * (Joutcyt*Out - Jcytout*Cyto) - Lys_area *
(Jcytlys*Cyto - Jlyscyt*Lyso));
dxdt_Lyso = 1/Lys_Volume * (Lys_area * (Jcytlys*Cyto - Jlyscyt*Lyso));
double Lys_pH = Lys_pHinside + (Lyso/ Buff_cap);
'
#==========================================================================#
                                  # END #
#==========================================================================#
```
